# Increased Macrophages and C1qA, C3, C4 Transcripts in the Midbrain of People With Schizophrenia

**DOI:** 10.3389/fimmu.2020.02002

**Published:** 2020-09-29

**Authors:** Tertia D. Purves-Tyson, Kate Robinson, Amelia M. Brown, Danny Boerrigter, Helen Q. Cai, Christin Weissleder, Samantha J. Owens, Debora A. Rothmond, Cynthia Shannon Weickert

**Affiliations:** ^1^Schizophrenia Research Laboratory, Neuroscience Research Australia, Sydney, NSW, Australia; ^2^School of Psychiatry, Faculty of Medicine, University of New South Wales, Sydney, NSW, Australia; ^3^Department of Neuroscience and Physiology, Upstate Medical University, Syracuse, NY, United States

**Keywords:** schizophrenia, postmortem, substantia nigra, midbrain, macrophage, CD163, ICAM1, complement

## Abstract

Increased cytokine and inflammatory-related transcripts are found in the ventral midbrain, a dopamine neuron-rich region associated with schizophrenia symptoms. In fact, half of schizophrenia cases can be defined as having a “high inflammatory/immune biotype.” Recent studies implicate both complement and macrophages in cortical neuroinflammation in schizophrenia. Our aim was to determine whether measures of transcripts related to phagocytosis/macrophages (CD163, CD64, and FN1), or related to macrophage adhesion [intercellular adhesion molecule 1 (ICAM1)], or whether CD163+ cell density, as well as protein and/or gene expression of complement pathway activators (C1qA) and mediators (C3 or C4), are increased in the midbrain in schizophrenia, especially in those with a high inflammatory biotype. We investigated whether complement mRNA levels correlate with macrophage and/or microglia and/or astrocyte markers. We found CD163+ cells around blood vessels and in the parenchyma and increases in ICAM1, CD163, CD64, and FN1 mRNAs as well as increases in all complement transcripts in the midbrain of schizophrenia cases with high inflammation. While we found positive correlations between complement transcripts (C1qA and C3) and microglia or astrocyte markers across diagnostic and inflammatory subgroups, the only unique strong positive correlation was between CD163 and C1qA mRNAs in schizophrenia cases with high inflammation. Our study is the first to suggest that more circulating macrophages may be attracted to the midbrain in schizophrenia, and that increased macrophages are linked to increased complement pathway activation in tissue and may contribute to dopamine dysregulation in schizophrenia. Single-cell transcriptomic studies and mechanistic preclinical studies are required to test these possibilities.

## Introduction

Dopaminergic dysregulation is at the core of schizophrenia symptoms, and includes subcortical hyperdopaminergia, underlying psychosis, and frontal hypodopaminergia, contributing to cognitive deficits (1, 2). Neuropathology is evident at the molecular and neurotransmitter level within the ventral midbrain of people with schizophrenia (3–8), the region where the majority of dopamine cell bodies are found. Increases in pro-inflammatory cytokines and gliosis (9) are some of the most robust pathological changes found in the midbrain of people with schizophrenia to date; however, the inflammatory mechanisms of midbrain inflammation that may contribute to dopamine dysregulation in patients are poorly understood.

A link between schizophrenia and the immune system is found using multiple strategies. Postmortem studies show increases in glial markers in the brain and higher expression of inflammatory mediators and cytokines in several brain areas, and increased inflammatory molecules are reported in the peripheral blood of people with schizophrenia (9–20). Importantly, the increases in cytokine transcripts are most obvious in a subset of schizophrenia cases, which we have termed a “high inflammatory biotype” or “high immune biotype” [e.g., (9, 12, 19, 20)]. High levels of inflammation are linked to more severe symptoms, greater neuropathology, and exacerbated cognitive deficits [e.g., (11, 18, 19, 21–24)]. Increased tissue inflammation can lead to increases in phagocytosis-primed macrophages in neurological disease and following brain injury (25–27); thus, we expect to find increased macrophages and higher complement production in the midbrain of those with schizophrenia, increased cytokines and gliosis (9).

In neuroinflammatory conditions, CD163+ macrophages infiltrate into brain tissue, including in encephalitis [both human immunodeficiency viral and simian immunodeficiency viral (HIV and SIV) encephalitis] (28, 29), multiple sclerosis (MS) (25), Parkinson's disease (PD), and Alzheimer's disease (AD) (26). Evidence suggests that macrophages may enter the brain in schizophrenia, as increases in the vascular macrophage capture molecule, intercellular cell adhesion molecule 1 (ICAM1) (30–32) and increases in CD163 mRNAs are found in the cortex and hippocampus (32–34). However, distinguishing macrophages from resident microglia is challenging due to overlap in expression of markers and due to the fact that macrophages can transform into microglial-like cells once they have entered the brain (35–38). One study, using direct RNA sequencing of microglia and peripheral (peritoneal-derived) macrophages from the same mice, determined that fibronectin 1 (FN1) is a macrophage-enriched gene not expressed in brain microglia and the hexosaminidase B (HEXB) (a cytoplasmic protein not associated with cellular activation) gene is uniquely and highly expressed by microglia in the brain (35). Here, we hypothesized that markers consistent with infiltrating macrophages (ICAM1, CD163, and FN1 mRNAs) would be increased in midbrain parenchyma near dopamine cell bodies in people with schizophrenia and a high inflammatory biotype. We previously reported increases in markers associated with microglial activation in the midbrain in schizophrenia, without a clear increase in microglia density (9). Here, we hypothesized that we would see no change in the pan marker of microglia (HEXB mRNA) but increases in gene expression of a marker typical of activated or pro-inflammatory microglia/macrophages (CD64, or FcgammaR1 mRNA). We also expected to detect no change or decreases in transcripts more often associated with anti-inflammatory microglia/macrophages (CD206 or mannose receptor C1, MRC1, mRNA) as the changes in the midbrain in schizophrenia appear to be more consistent with a tissue-damaging phenotype.

Along with being major sources of cytokines in the brain, microglia, astrocytes, and macrophages also produce complement protein (39, 40). The complement system, which interacts with both the adaptive and the innate immune systems to eliminate pathogens and damaged cells, is linked with development and maintenance of the CNS, as well as neurodevelopmental and psychiatric disorders including schizophrenia [reviewed in (41–46)]. The strongest genetic association in schizophrenia is with the major histocompatibility locus (MHC) (47), which includes increased copy number variation load within the complement 4 (C4) gene (48). C4 mRNA is also increased in multiple cortical regions in schizophrenia cases compared to non-schizophrenia controls (controls and bi-polar cases combined) (48). However, complement levels have not been investigated in the midbrain in schizophrenia. Here, we hypothesized that activators (C1qA) and/or mediators (C3 or C4) of the classical pathway (49) would be elevated in the midbrain of those with schizophrenia compared to controls, especially in those with a high inflammatory biotype. Additionally, we hypothesized that there may be a change in mRNA encoding neuroprotective or “don't eat me” signals in the midbrain of people with schizophrenia that attempt to attenuate or block complement [CD55 or DAF (decay accelerating factor) and CD59 or membrane attack complex inhibitory protein (MAC-IP)] (49). We also hypothesized that complement mRNA levels would be positively associated with infiltrating macrophage, and/or with microglia and/or astrocyte [glial fibrillary acidic protein (GFAP)] markers.

## Methods

### Human Postmortem Tissue Collection and Midbrain Cohort Demographics

Human tissue experiments were approved by the University of New South Wales Human Research Ethics Committee (HREC #17826). Midbrain tissue was provided by the New South Wales Brain Tissue Resource Center (30/30 control/schizophrenia cases) and processed as previously described to generate mRNA and protein midbrain cohorts (7). The final midbrain mRNA cohort in this manuscript comprised 28 schizophrenia cases and 28 controls and the final midbrain protein cohort comprised 26 schizophrenia cases and 28 controls (Table 1A). In both protein and mRNA cohorts, diagnostic groups were matched for age, gender, and postmortem interval (PMI) and in the mRNA cohort, diagnostic groups were also matched for RNA integrity number (RIN). All schizophrenia patients received antipsychotic medication.

**Table 1A:** Demographic details of the postmortem midbrain mRNA and protein cohorts classified by diagnosis.

**Demographics**	**mRNA cohort**			**Protein cohort**		
	**Control (28)**	**Schizophrenia (28)**	**Statistics**	**Control (28)**	**Schizophrenia (26)**	**Statistics**
Age (years)	50.54	51.36	*t*_(54)_ = −0.27	52.21	52.29	*t*_(52)_ = −0.23,
	(22–67)	(26–67)	*p* = 0.79	(22–69)	(26–67)	*p* = 0.82
Gender (M, F)	(19,9)	(20,8)	–	(19,9)	(10, 26)	–
pH	6.66 ± 0.26	6.51 ± 0.20	***t***_**(54)**_ **= 2.52**	6.69 ± 0.24	6.51 ± 0.23	***t***_**(52)**_ **= 2.62**,
			***p*** = 0.015^*^			***p*** = 0.011^*^
PMI (hours)	31.68 ± 10.21	35.66 ± 17.71	*t*_(54)_ = −1.03	32.75 ± 9.90	38.21 ± 18.20	*t*_(52)_ = −1.38,
(range)	(15–50)	(5–72)	*p* = 0.31	(15–50)	(5–72)	*p* = 0.17
RIN	5.56 ± 1.15	5.61 ± 1.31	*t*_(54)_ = −0.15	–	–	***–***
			*p* = 0.88			
Duration of illness	–	28.31 ± 12.72	–	–	29.12 ± 13.02	–
		(4–49)			(4–49)	
Daily CPZ (mg)	–	736.45 ± 520.50	–	–	716.37 ± 557.76	–
Last recorded CPZ (mg)	–	597.54 ± 497.64	–	–	616.20 ± 506.60	–
Lifetime CPZ (g)	–	8231.44 ± 8714.24	–	–	8427.92 ± 9348.47	–

*F, female; M, male; PMI, postmortem interval; RIN, RNA integrity; SCZ, schizophrenia, Data are mean ± s.d. Age and PMI ranges are in brackets. Bold values indicate significant changes. Note the group sizes are different in the cohorts as different cases were excluded due to either poor quality RNA and/or protein. A single schizophrenia case in this mRNA cohort was lost from the mRNA cohort published previously (7) due to failure of cDNA synthesis*.

* *p < 0.05*.

Midbrain cases were categorized into low or high inflammatory subgroups using a two-step recursive cluster of seven inflammatory-related transcripts in the entire midbrain cohort (cases and controls) as described previously (9). Briefly, missing transcript values were replaced using an expectation maximization algorithm in order to retain as many cases as possible. After defining the potential predictors, the overall model quality needed to exceed 0.5 (on a scale of 0–1.0), with predictors of least importance removed until all predictors contributed significantly to the model (predictor importance >0.4 on a scale of 0–1.0). The clustering algorithm revealed 13 individuals in a high inflammatory cluster and 44 individuals in a low inflammatory cluster. The high inflammatory subgroup was defined by high SERPINA3, IL6, IL1β, and TNFα mRNAs. The 13 cases in the high inflammatory subgroup were all schizophrenia cases (high inflammatory/schizophrenia) and the remaining 15 schizophrenia cases had low expression of inflammatory transcripts (low inflammatory/schizophrenia), as did all control cases (low inflammatory/control) and the distribution of high and low inflammatory cases in the diagnostic groups was significantly different (χ^2^ = 57.0, *p* < 0.0001, *N* = 57). There was an equivalent distribution of males and females between the diagnosis/inflammatory subgroups (χ^2^ = 0.54, *p* = 0.76, *N* = 56). Additionally, no differences in gene/protein expression according to sex were identified by *t*-tests (data not shown, all *p* > 0.05).

Demographics for the midbrain mRNA and protein cohorts by inflammatory subgroup are shown in Table 1B. Final cases included in the mRNA and protein cohorts do not completely overlap, as some cases had poor protein and/or mRNA quality (2–4 cases per group).

**Table 1B:** Demographic details of the postmortem midbrain mRNA and protein cohorts classified by inflammatory subgroup.

**Demographics**	**mRNA cohort**				**Protein cohort**			
	**Control (28)**	**SCZ low inflammatory (15)**	**SCZ high inflammatory (13)**	**Statistics**	**Control (26)**	**SCZ low inflammatory (13)**	**SCZ high inflammatory (12)**	**Statistics**
Age (years)	50.54	48.27	54.92	χ2(2) = 2.81, *p* = 0.25	51.19	49.31	56.17	χ2(2) = 2.89, *p* = 0.24
	(22–67)	(30–64)	(26–67)		(22–67)	(30–64)	(26–67)	
Gender (M, F)	(20,8)	(11,4)	(8,5)	–	(19,7)	(9,4)	(7,5)	–
pH	6.66 ± 0.26	6.52 ± 0.20	6.49 ± 0.20	***χ*****2(2) = 8.51**, ***p*** = 0.014^*^	6.69 ± 0.22	6.54 ± 0.20	6.52 ± 0.18	***F***_**(2, 48)**_ **= 4.02**, ***p*** = 0.024^*^
PMI (hours)	31.68 ± 10.21	33.43 ± 14.66	38.23 ± 21.01	*F*_(2, 53)_ = 0.91, *p* = 0.41	33.42 ± 10.21	35.19 ± 14.93	39.62 ± 21.30	*F*_(2, 48)_ = 1.00, *p* = 0.38
(range)	(15–50)	(18–64)	(5–72)		(15–50)	(18–64)	(5–72)	
RIN	5.56 ± 1.15	5.53 ± 1.28	5.70 ± 1.39	*F*_(2, 53)_ = 0.079, *p* = 0.92	–	–	–	–
Duration of illness	–	26.43 ± 11.65	31.62 ± 13.926	*t*_(26)_ = −1.073, *p* = 0.29	–	25.92 ± 11.30	32.33 ± 14.29	*t*_(22)_ = −1.220, *p* = 0.24
Daily CPZ (mg)	–	483.54 ± 177.63	1039.93 ± 637.06	***t***_**(10.169)**_ **= 2.68**, ***p*** = 0.023^*^	–	422.30 ± 116.23	1043.11 ± 675.69	***U*** **= 81.0**, ***p*** = 0.002^***^
Last recorded CPZ (mg)	–	361.53 ± 316.56	869.85 ± 538.82	***U*** **= 156.5**, ***p*** = 0.005^**^	–	391.00 ± 331.35	860.17 ± 561.60	***U*** **= 210.5**, ***p*** = 0.019^*^
Lifetime CPZ (g)	–	4204.952 ± 2170.41	13063.22 ± 11129.88	***U*** **= 100.0**, ***p*** = 0.007^**^	–	3799.83 ± 1800.43	13570.25 ± 11681.88	***U*** **= 77.0**, ***p*** = 0.008^**^

*F, female; M, male; PMI, postmortem interval; RIN, RNA integrity; SCZ, schizophrenia, Data are mean ± s.d. Age and PMI ranges are in brackets. Bold values indicate significant changes. ∧Control group sizes are different in the cohorts as there is no inflammatory biotype for 2 cases that were included in the protein cohort but excluded from the mRNA cohort due to poor qPCR data*.

* *p < 0.05*,

** *p < 0.01*,

*** *p < 0.001*.

### RNA Extraction and Quantitative Real-Time PCR

Total RNA was extracted from human substantia nigra using TRIzol and cDNA was synthesized using Superscript IV (Life Technologies, Scoresby, Australia). RNA quality was determined using the Agilent Bioanalyzer 2100 (Agilent Technologies, Santa Clara, CA) and cases with low RINs were excluded (1 control, 2 schizophrenia). Quantitative PCR was conducted with either the Applied Biosystems Prism 7900HT Fast Real Time system or with the high-throughput Fluidigm Biomark^TM^ HD system (Fluidigm, San Francisco, CA, USA) at the Ramaciotti Center for Genomics, UNSW, Sydney, Australia. Expression of genes measured on both platforms are highly correlated (e.g., tyrosine hydroxylase, β-actin, and ubiquitin-C gene expression, all *r* > 0.70, *p* < 0.05).

TaqMan gene expression assays were used (Life Technologies, Australia, Supplementary Table 1). Housekeeper controls (β-actin, TATA-Box-binding protein, ubiquitin C, and glyceraldehyde 3-phosphate dehydrogenase mRNAs) and the geomean of all four housekeepers did not differ according to diagnosis by qPCR measured by high throughput Fluidigm (all *t* < 1.16, df = 53–54, *p* > 0.05) (7). Serial dilutions of pooled cDNA from all samples were included for quantitation of sample expression by the relative standard curve method. Gene expression data was analyzed with SDS 2.4 software (ABI, Life Technologies) or Fluidigm Real-Time PCR Analysis software version 4.5.2. Relative gene expression was normalized to the geomean of the four housekeepers. Gene expression data are presented as relative mRNA levels ± SEM.

### Protein Extraction and Western Blotting

Midbrain protein samples were prepared as previously described (7). Briefly, samples were homogenized in 0.1 M Tris (pH 7.5), 50% glycerol, proteinase inhibitor cocktail and aprotinin (0.015 mM) (all Sigma-Aldrich, Castle Hill, Australia) using a handheld electric homogenizer (Polytron, Kinematica, Lucerne, Switzerland). Protein was quantified using a Bradford protein assay (Sigma-Aldrich).

Protein, diluted in 2x Laemmli Buffer (CD163 and C4) or NuPAGE Sample Buffer (C3), was separated on SDS-polyacrylamide gels (BioRad or Thermo Fisher, Melbourne, Australia) and transferred onto nitrocellulose membranes (0.45 μm). Several randomly selected samples were pooled and an aliquot was run on all gels for standardization between blots (internal control). Nitrocellulose membranes were blocked in 5% skim milk in tris-buffered saline with Tween-20 (TBST). Membranes were incubated overnight at 4°C in primary antibody diluted in 1% skim milk in TBST. Membranes were washed and incubated with horseradish peroxidase-conjugated (HRP) secondary antibodies in 1% skim milk in TBST for 1 h at room temperature. All membranes were washed in TBST and re-probed with mouse anti-β-actin (1:5,000; MAB1501, Millipore) for at least 2 h at 4°C, followed by incubation with an HRP-goat anti-mouse secondary antibody.

Immunoreactive protein bands were visualized using enhanced chemiluminescence reagent (Millipore). CD163 membranes were exposed to radiographic film (GE Healthcare, IL) which were scanned, and band densities converted to numerical values using ImageJ software (ImageJ, National Institutes of Health, USA). C3 and C4 membranes were captured on an iBright 1500 Imaging System (Thermo Fisher Scientific) and band densities determined using iBright Analysis Software (V3.0.1, Thermo Fisher Scientific). Band density of CD163, C3, and C4 for each sample was normalized to the corresponding band density of β-actin detected in the same lane and then normalized to an internal control that was loaded onto each gel for standardization between blots and expressed as relative protein expression levels ± SEM.

For CD163, 15 μg midbrain protein homogenate was run on an 8% Bis-Tris acrylamide gel and CD163 was detected with a rabbit anti-CD163 primary antibody (1:400, ab182422, Abcam, Melbourne, Australia) and a goat anti-rabbit HRP-conjugated secondary antibody (1:2,000, AP132P, Millipore, Sydney, Australia). Mouse thymus protein homogenate (ab29285, Abcam, 2 μg) was used as a positive control for CD163 protein. For complement proteins, 10 μg midbrain protein homogenate was run on a 3–8% NuPage Tris-Acetate precast acrylamide gel (C3 detection) (Thermo Fisher, USA) or an 8% Bis-Tris acrylamide gel (C4 detection). C3 and C4 proteins were detected with rabbit anti-complement primary antibody (C3, 1:3,000, ab97462; C4, 1:3,000, ab173577, both Abcam) and a goat anti-rabbit HRP-conjugated secondary antibody (1:10,000, AP307P Millipore). Molecular weight of immunoreactive bands were determined by comparison to a molecular weight marker (Precision Plus Protein standards, #1610374, Biorad).

### Identification of Parenchymal Macrophages by Immunohistochemistry

Fresh frozen midbrain sections (14 μm) were used for 3,3 diaminobenzidine (DAB) immunohistochemistry to detect location and to count density of CD163+ cells, and double-label immunofluorescence was used to detect cells labeled with CD163 and/or IBA1. Negative control slides, incubated in the absence of primary antibodies, were included in all experiments.

For DAB immunohistochemistry, two midbrain sections from each case were fixed in 4% paraformaldehyde (PFA) and treated with 0.75% H_2_O_2_ and 75% methanol for 20 min to inhibit endogenous peroxidase activity. Slides were blocked in 10% normal horse serum (Vector Laboratories, Burlingame, CA) and incubated (4°C overnight) with mouse anti-CD163 primary antibody (1:400, ab111250, Abcam). Tissue was washed with phosphate buffered saline (PBS) and incubated in horse anti-mouse IgG biotinylated secondary antibody (1:500, BA-2000, Vector Laboratories) before conjugation with avidin-peroxidase complex (VectaStain kit PK-4000, Vector Laboratories). Antibody binding was visualized with DAB (Sigma-Aldrich), washed, dehydrated, stained with Nissl (5 min exposure to 0.02% thionin), dehydrated in ethanol, cleared in xylene and cover slipped with Permount (Fisher Scientific, Pittsburgh, PA). Density of CD163+ cells was examined in two dimensions with a 20X objective (Nikon Eclipse 80i light microscope, Coherent Scientific, Hilton, SA, Australia). Stereo-Investigator (v23, MicroBrightField, Williston, VT) was used to randomly place two grids containing twelve 115 × 115 μm boxes in the substantia nigra, identified by the presence of neuromelanin containing cells ventral to the red nucleus and referencing tyrosine hydroxylase stained slides from the same case. CD163+ cells were counted in six randomly placed boxes of each of the two 115 × 115 μm grids, with two permissive and two non-permissive edges (total area counted was 158.7 μm^2^). Counting was performed blind to diagnosis, and CD163+ cell number calculated as the total number of CD163+ cells divided by the total area counted and cell density expressed as cells/mm^2^.

For double-label immunofluorescence, two midbrain tissue sections each from three high inflammatory schizophrenia cases, were fixed in 4% PFA (Sigma-Aldrich) for 10 min at 4°C and blocked for 1 h in 10% normal donkey serum (Millipore, Temecula, CA, USA) and 0.3% Triton X-100 (Sigma-Aldrich) in PBS for 1 h. Slides were incubated overnight at 4°C with mouse anti-CD163 (1:250, ab111250, Abcam) and rabbit anti-IBA1 (1:200, #111583, NovoPro, Shanghai, China) antibodies in 0.3% Triton X-100 and 0.05% BSA in PBS. Slides were washed 3 × 10 min in PBS and Alexa Fluor-labeled secondary antibodies (Alexa 594 anti-mouse; Alexa 488 anti-rabbit, both 1:500; Thermo Fisher) in buffer as above, were added for 1 h at room temperature. To minimize auto-fluorescence, slides were washed in 15 mM cupric sulfate (Sigma-Aldrich) and 50 mM ammonium acetate (Sigma-Aldrich) for 2 × 15 min. Slides were counterstained with 1:1,000 DAPI (Sigma-Aldrich) in PBS and cover-slipped with anti-fade mounting media (ProSciTech, Kirwan, Australia). Co-localization of CD163 and IBA1 immunofluorescent signal was analyzed by acquiring *z* stacks of up to 20 images with a LSM800 Zeiss confocal microscope (Zeiss Australia, Lonsdale, AUS), equipped with a high efficiency GaAsP detector, two multi-alkali photomultiplier tubes and the following objectives: 20x air objective with numerical aperture (NA) 0.75; 40x oil objective with NA 1.3, and 63x oil objective with NA 1.4. The system is equipped with 405, 488, 561, and 640 nm lasers as the excitation sources. Detection wavelengths for double labeling immunofluorescence were 510–595 nm for AF488, 595–700 nm for AF594, and 410–510 nm for DAPI. All *z* stacks were imaged with either 20x air (1.5 zoom), 40x oil objective (1.0 zoom), or 63x oil objective (0.5 zoom). Maximum intensity projections were used to visualize the brightest voxel in the final image.

### Statistical Analyses

Statistical tests were performed using SPSS (V25, IBM, Armonk, NY, USA) and statistical significance was set at *p* < 0.05.

#### Cohort Demographic Variables and mRNA Cohort Housekeepers

Demographic variables and antipsychotic exposure (daily, lifetime, last dose chlorpromazine equivalents [CPZ]) for the midbrain mRNA and protein cohorts are shown by diagnosis in Table 1A and by diagnosis/inflammatory subgroups in Table 1B. Mann-Whitney or Kruskal-Wallis tests were performed to detect differences in non-normally distributed demographic variables in the mRNA (age, pH) and protein (age) cohorts in the diagnosis/inflammatory subgroups. Analysis of variance (ANOVA) was used to detect differences in normally distributed demographic variables in the diagnosis/inflammatory subgroups (RIN in the mRNA cohort, pH in the protein cohort) and to confirm no change in individual housekeeper gene expression, the geomean of the four housekeepers or the β-actin protein expression levels between diagnosis/inflammatory subgroups (all *t* < 1.71 df = 51–52, *p* > 0.05). Differences in daily CPZ, last recorded CPZ and lifetime CPZ between the schizophrenia/low and schizophrenia/high inflammatory subgroups (explored with independent samples *t*-test or Mann-Whitney *U*-test) were previously reported (9) and are shown in Table 1B.

#### Analysis of Relative Gene and Protein Expression and CD163+ Cell Density by Diagnosis and by Diagnosis/Inflammatory Subgroup

Outliers were defined by being outside two standard deviations of the mean by diagnostic group and by diagnosis/inflammatory subgroup (0–3 values were identified and removed per group per gene of interest). Normality was examined with the Shapiro-Wilk test. If data was not normal in any group, the raw normalized data was log_10_ transformed, measurements outside two standard deviations were removed, and data re-tested for normality to allow for parametric statistical tests. Pearson's correlations (PMI, RIN, and pH) and Spearman's correlations (age) between demographic variables and genes/proteins of interest were used to identify covariates. No gene or protein of interest correlated with PMI, whilst genes, but not proteins, often correlated with age, RIN, and pH (Supplementary Table 2). If no covariates were identified, data was analyzed using independent samples two-tailed *t*-tests (control vs. schizophrenia) or by ANOVA (inflammatory/diagnosis subgroups). In the case of CD163+ cell density, we had a directional hypothesis and one-tailed *t*-tests were performed. If a gene/protein of interest correlated with RIN or age, this demographic factor was included as a covariate in an analysis of covariance (ANCOVA). Brain pH was not included as a covariate even if it correlated with a gene/protein of interest as reduced pH is considered part of the disease state in schizophrenia and is related to inflammation (50). Least significant difference (LSD) *post-hoc* tests were performed to identify changes between each of the three groups.

#### Correlations Between Complement and Cell Marker Gene Expression

The relationships between complement-related gene expression and glia/immune cell marker mRNAs were explored between control and schizophrenia groups, and between low and high inflammatory schizophrenia subgroups. Given that these analyses are exploratory; all cases were considered (no outliers removed). Analysis was run separately on control/schizophrenia cases, or control/schizophrenia inflammatory subgroups using Pearson's correlations. Partial correlations were run if covariates were identified (Supplementary Table 2). Correlation coefficients in the groups compared statistically were converted to *z* scores using Fisher's *r* to *z* transformation and the *z*-test statistic reported.

#### Analysis of Antipsychotic Treatment and Pre- and Ante-Mortem Factors

Antipsychotic treatment was converted to lifetime, daily, and last recorded dose of CPZ equivalents as reported previously (7). Spearman's correlations were used to explore the relationship between antipsychotic treatment measures as well as duration of illness (in years), and genes/proteins of interest (Table 2).

**Table 2:** Correlations between mRNA transcripts, proteins of interest, chlorpromazine equivalents, and duration of illness (in schizophrenia cases only).

**Gene of interest**	**Chlorpromazine equivalent measure**	***N***	**Correlation coefficient**	***p***
CD163 mRNA	Lifetime	22	**0.429**	0.047^*^
	Mean daily	22	**0.506**	0.016^*^
	Last dose	28	0.246	0.207
	Illness duration	28	0.250	0.218
ICAM1 mRNA	Lifetime	22	**0.524**	0.012^*^
	Mean daily	22	**0.581**	0.005^**^
	Last dose	28	0.198	0.311
	Illness duration	28	0.227	0.245
CD64 mRNA	Lifetime	22	0.339	0.123
	Mean daily	22	**0.538**	0.010^*^
	Last dose	28	0.303	0.117
	Illness duration	28	0.116	0.558
MRC1 mRNA	Lifetime	22	−0.045	0.844
	Mean daily	22	−0.014	0.950
	Last dose	28	−0.174	0.375
	Illness duration	28	0.279	0.151
FN1 mRNA	Lifetime	22	0.090	0.689
	Mean daily	22	0.217	0.333
	Last dose	28	−0.057	0.772
	Illness duration	28	0.023	0.909
HEXB mRNA	Lifetime	22	0.061	0.787
	Mean daily	22	−0.150	0.506
	Last dose	28	−0.292	0.131
	Illness duration	28	0.246	0.208
C1qA mRNA	Lifetime	22	**0.432**	0.045^*^
	Mean daily	22	**0.672**	0.001^***^
	Last dose	28	0.121	0.539
	Illness duration	28	0.158	0.423
C3 mRNA	Lifetime	22	0.264	0.235
	Mean daily	22	**0.506**	0.016^*^
	Last dose	27	0.123	0.534
	Illness duration	27	0.190	0.334
C4 mRNA	Lifetime	22	0.403	0.063^#^
	Mean daily	22	**0.488**	0.021^*^
	Last dose	28	0.280	0.148
	Illness duration	28	0.051	0.798
CD59 mRNA	Lifetime	22	0.328	0.137
	Mean daily	22	0.285	0.198
	Last dose	28	−0.085	0.668
	Illness duration	28	0.289	0.135
CD55 mRNA	Lifetime	22	0.237	0.288
	Mean daily	22	0.232	0.298
	Last dose	28	−0.036	0.855
	Illness duration	28	0.308	0.111
CD163 protein	Lifetime	19	**0.498**	0.030^*^
	Mean daily	19	0.434	0.063^#^
	Last dose	25	0.294	0.154
	Illness duration	24	0.341	0.103
C3 protein 125 kDa	Lifetime	18	−0.444	0.065^#^
	Mean daily	18	−0.442	0.067^#^
	Last dose	24	−0.172	0.422
	Illness duration	23	−0.102	0.644
C3 protein 43 kDa	Lifetime	18	−0.370	0.131
	Mean daily	18	−0.377	0.123
	Last dose	24	−0.236	0.267
	Illness duration	23	0.080	0.718
C4 protein 92 kDa	Lifetime	18	−0.095	0.708
	Mean daily	18	−0.330	0.181
	Last dose	24	−0.312	0.137
	Illness duration	23	0.010	0.962

*Bold values denote significant correlations*.

# *p < 0.1*,

* *p < 0.05*,

** *p < 0.01*,

*** *p < 0.001*.

Student's two-tailed *t*-tests (equal variances not assumed) were used to explore differences in gene/protein expression levels between schizophrenia cases using clozapine at time of death (indicative of possible treatment resistance) and those that were on other antipsychotics, between those on predominantly first generation, predominantly second generation, or a combination of antipsychotic types. The effect of mostly positive or negative symptoms was also considered. We tested for differences in gene/protein expression according to depression, smoking at death, smoking throughout lifetime and suicide (in cases where data was available). These analyses were considered exploratory as the group sizes are small.

## Results

### Increased ICAM1 Gene Expression in the Midbrain of Schizophrenia Cases With a High Inflammatory Biotype

**Figure 1:**
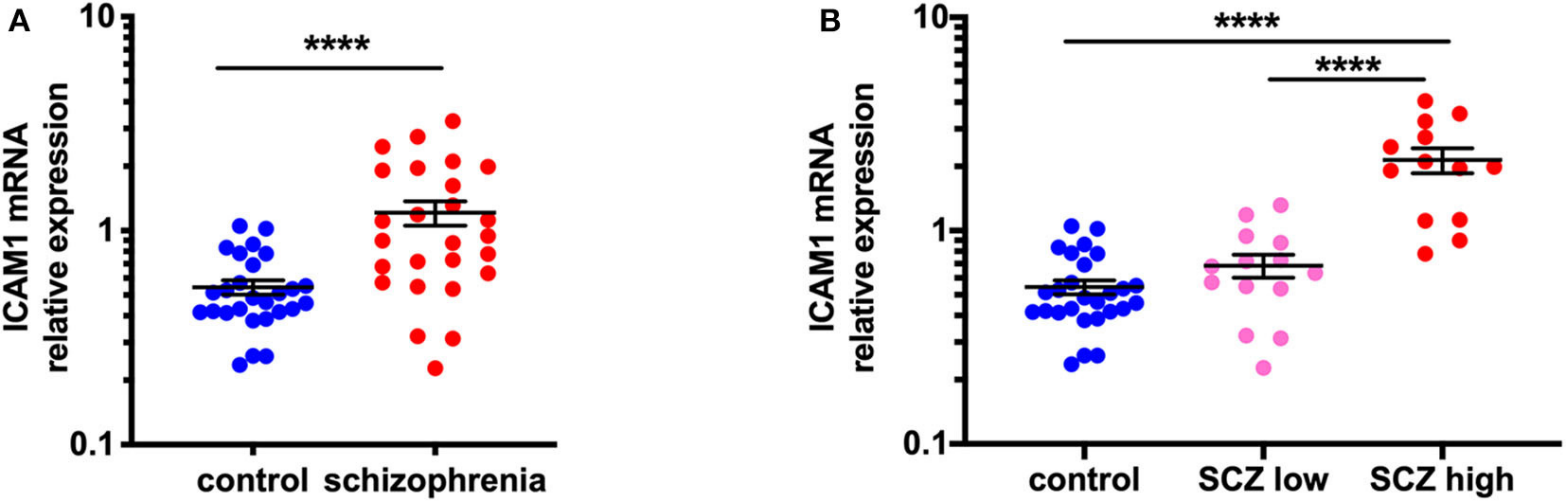
ICAM1 gene expression is increased in the midbrain in schizophrenia cases with a high inflammatory biotype. **(A)** Gene expression of ICAM1 was increased in the midbrain of schizophrenia cases compared to controls (*t* = −5.25, df = 40.40, *p* < 0.0001). **(B)** Analysis by inflammatory subgroup revealed this increase was in the subgroup of schizophrenia cases with a high inflammatory biotype (*F* = 39.84, df = 51,2, *p* < 0.0001). Data are mean ± SEM, ****p < 0.0001. SCZ low, low inflammatory/schizophrenia; SCZ high, high inflammatory/schizophrenia.

We found that ICAM1 mRNA was increased by 123.3% in the midbrain when all schizophrenia cases were compared to controls (*t* = −5.25, df = 40.40, *p* < 0.0001) (Figure 1A). When analyzed by inflammatory subgroup, ICAM1 gene expression was increased in the high inflammatory/schizophrenia subgroup by 213.4 and 295.1% compared to the low inflammatory/schizophrenia subgroup and the control group, respectively (*F* = 39.84, df = 51,2, *p* < 0.0001, both comparisons *p* < 0.0001) (Figure 1B). We did not detect a difference in ICAM1 mRNA in the low inflammatory/schizophrenia group compared to the control group (*p* > 0.05).

### Increased CD163 Macrophage Marker—mRNA and Protein Levels—in the Midbrain in Schizophrenia Is Exacerbated in Cases Classified With a High Inflammatory Biotype

**Figure 2:**
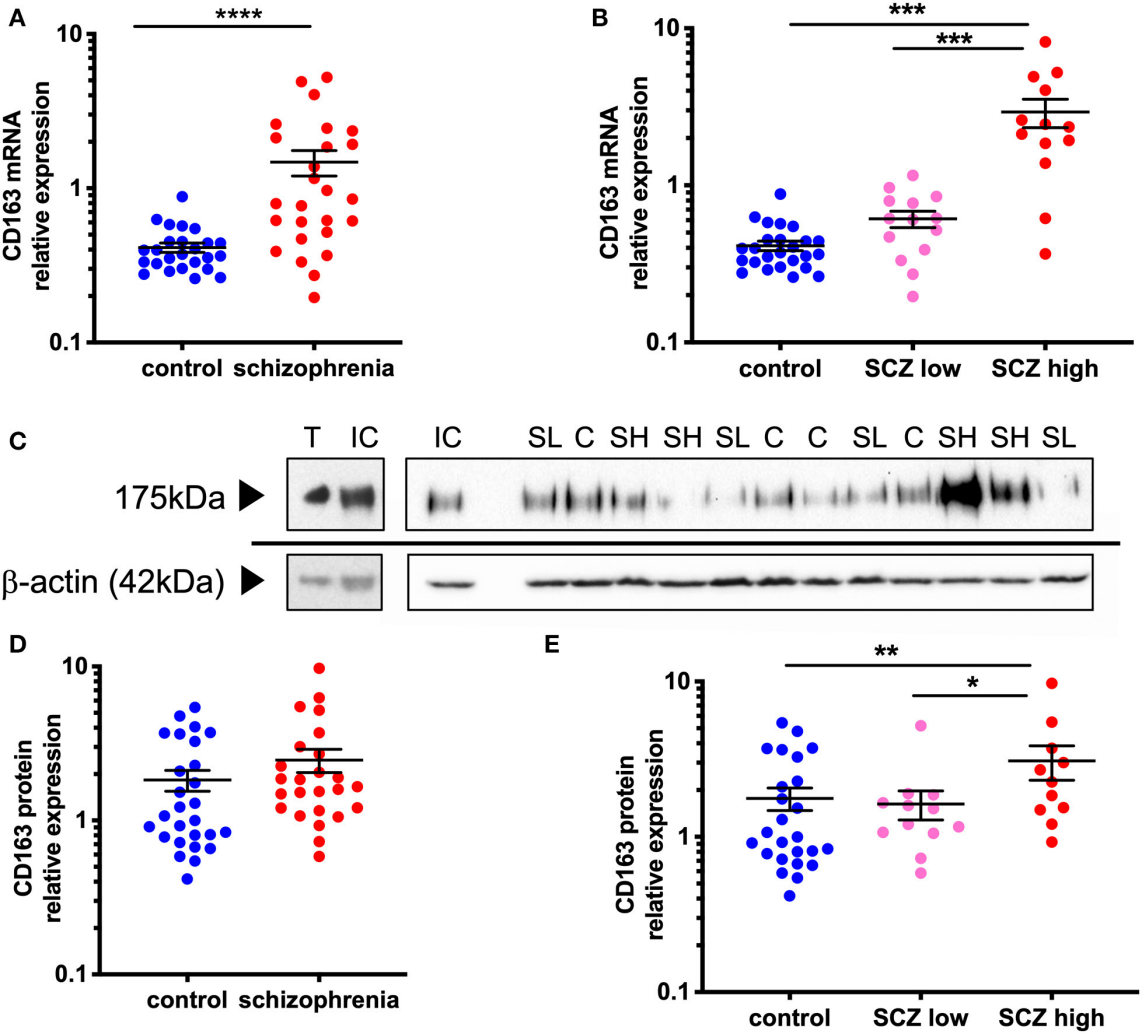
CD163 mRNA and protein is increased in the midbrain in schizophrenia cases classified with a high inflammatory biotype. **(A)** CD163 mRNA was significantly increased in the midbrain of schizophrenia cases compared to control cases (*F* = 25.31 df = 49,1, *p* < 0.001). **(B)** CD163 mRNA was significantly increased in the high inflammatory/schizophrenia subgroup compared to both the low inflammatory/schizophrenia subgroup and the controls (*F* = 35.18, df = 50,2, *p* < 0.001). **(C)** CD163 band at 175 kDa was detected in all samples including in mouse thymus positive control. β-actin (~42 kDa) was detected in all samples and used as a normalizing control. **(D)** CD163 protein expression was not significantly different in schizophrenia cases compared to controls (*t* = −1.63, df = 50, *p* = 0.11) but, when analyzed by **(E)** inflammatory subgroup (*F* = 4.65, df = 46,2, *p* = 0.014), CD163 protein was significantly increased in the high inflammatory/schizophrenia subgroup compared to both the low inflammatory/schizophrenia subgroup and the controls. Data are mean ± SEM, *p < 0.05, **p < 0.01, ***p < 0.001, ****p < 0.0001. C, control; SL, low inflammatory/schizophrenia; SH, high inflammatory/schizophrenia; IC internal control; T, thymus.

In support of our hypothesis, we saw increased gene expression and protein levels of the macrophage marker, CD163, in the region containing the dopamine cell bodies in schizophrenia, and these measures were most robustly increased in the schizophrenia cases with a high inflammatory biotype. When comparing by diagnosis alone, CD163 mRNA was increased by 255.7% (*F* = 25.31, df = 49,1, *p* < 0.001) in schizophrenia cases relative to controls (Figure 2A). When analyzed by diagnosis/inflammatory subgroups (*F* = 35.18, df = 50,2, *p* < 0.001) (Figure 2B), it was apparent that the significant increase in CD163 in the schizophrenia group was driven by the high inflammatory/schizophrenia group which had 562.6 and 610.9% more CD163 mRNA than the low inflammatory/schizophrenia and the control subgroups, respectively (both *p* < 0.001). The low inflammatory/schizophrenia subgroup was not significantly different from the control group (*p* > 0.05).

Western blotting revealed a 175 kDa anti-CD163 immunoreactive band in all human midbrain cases, consistent with the anti-CD163 immunoreactive band size in the mouse thymus (used as a positive control, Figure 2C). CD163 protein levels generally reflected the changes found in gene expression, however at a reduced magnitude, which was expected from this less quantitative technique. Although CD163 protein was increased by 34.8% in the midbrain in schizophrenia cases relative to controls, this diagnostic comparison did not reach statistical significance (*t* = −1.63, df = 50,1, *p* = 0.11) (Figure 2D). However, we found that the high inflammatory/schizophrenia subgroup had a significant (89.6 and 74.3%) increase in CD163 protein levels compared to the low inflammatory/schizophrenia and control subgroups, respectively (*F* = 4.65, df = 46,2, *p* = 0.014, both comparisons *p* < 0.05, Figure 2E).

### CD163+ Macrophages Are in Proximity to Dopaminergic Neurons in Substantia Nigra in Schizophrenia and Control Brains and Are Increased in Density in Schizophrenia Cases

**Figure 3:**
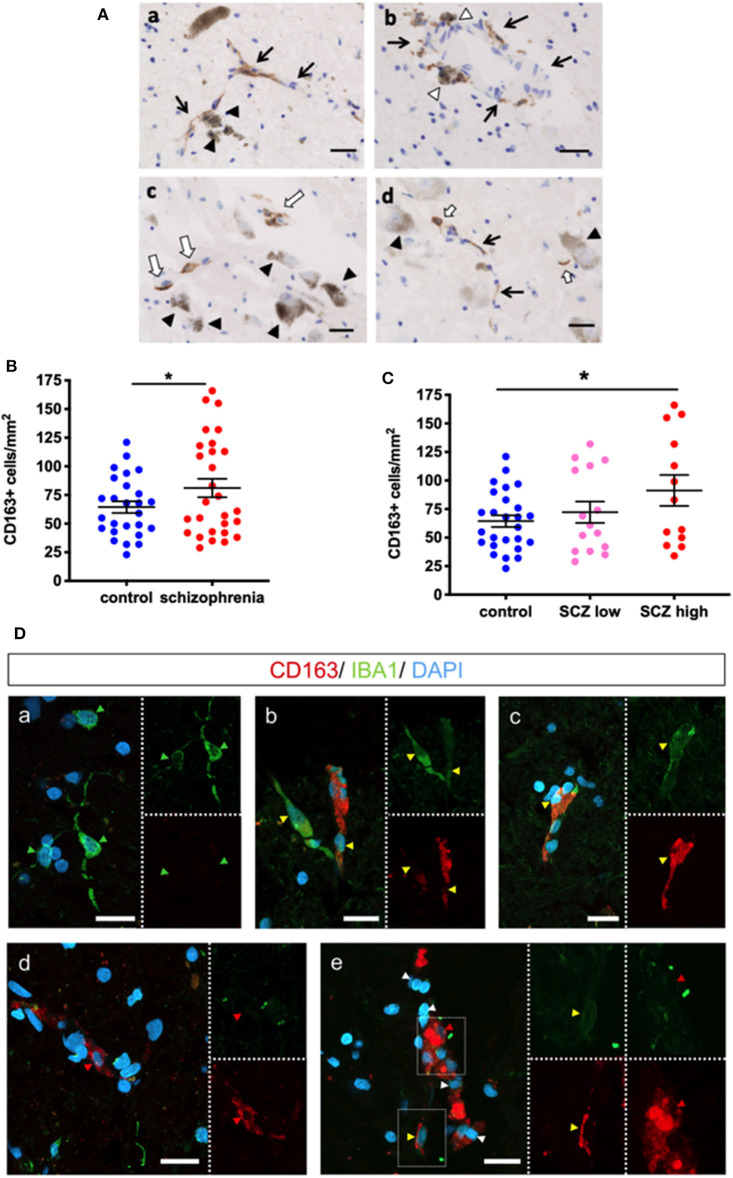
CD163 immunoreactive cells are in the parenchyma and in close proximity to dopamine neurons in the substantia nigra. **(A)** Dopaminergic neurons are identified by dark brown neuromelanin (black triangles). Black arrows indicate blood vessels. (a) CD163+ cells associated with blood vessels are in close proximity to dopaminergic neurons. (b) CD163+ cells are observed on the parenchymal side of blood vessels (white triangle). (c,d) CD163+ cells not associated with vessels (white arrows) are in close proximity to dopaminergic neurons (black triangle). Scale bar = 20 μm. **(B)** The number of CD163+ cells was increased when analyzed by diagnosis (*t* = 1.71, df = 52, *p* = 0.047) and **(C)** was increased in the high inflammatory/schizophrenia group by planned *post-hoc* comparison (*p* = 0.030). **(D)** IBA1 (green) and CD163 (red) expression in cells in the midbrain. (a) shows IBA1+/CD163- microglia (green arrowheads) in the midbrain. (b) Two IBA1+/CD163+ cells (yellow arrowheads), one (left cell) with strong positivity for IBA1 and low expression of CD163, and one (right cell) with strong CD163 expression and low IBA1 expression. (c) Another double-labeled microglia/macrophage (yellow arrowhead). (d) An IBA1-/CD163+ putative macrophage (red arrowhead). (e) IBA1-/CD163+ (red arrowhead) and IBA1+/CD163+ cells (yellow arrowhead). Nuclei are stained with DAPI (blue). Scale bars = 20 μm. Data are mean ± SEM, *p < 0.05.

CD163+ cells were found in the midbrain in proximity to melanin-containing dopaminergic neurons in both control and schizophrenia cases (Figure 3A). In the substantia nigra, 64.46 ± 5.09 and 81.11 ± 8.09 CD163+ cells/mm^2^ were counted in the controls and schizophrenia cases, respectively. When we tested if there was an increase in CD163+ cell density, we found a statistically significant increase in density of cells consistent with macrophages in people with schizophrenia compared to controls (*t* = 1.71, df = 52, *p* = 0.047) (Figure 3B). When density was examined according to inflammatory subgroup, we found 72.27 ± 9.35 CD163+ cells/mm^2^ in the low inflammatory/schizophrenia subgroup, and 91.31 ± 13.54 CD163+ cells/mm^2^ in the high inflammatory/schizophrenia subgroup (which was significantly different from the control group [*p* = 0.030] by planned *post-hoc* comparison) (Figure 3C).

### Cumulative Evidence Suggests That the CD163+ Cells Are Infiltrating Macrophages

We sought to distinguish between macrophages and resident microglia in the brain parenchyma with double-label fluorescence immunohistochemistry for CD163 and the pan-microglial marker IBA1 in the high inflammatory/schizophrenia cases. As expected, we found CD163-/IBA1+ cells, microglia, (Figure 3Da) throughout the parenchyma. We also identified CD163+/IBA1+ (high intensity) and CD163+/IBA1+ (low intensity) cells (Figures 3Db,c). We also identified CD163+/IBA1- cells in the midbrain parenchyma (Figure 3Dd) and in association with blood vessels (Figure 3De).

By diagnosis, gene expression of FN1, expressed by peripheral macrophages and not microglia, was increased in the midbrain in schizophrenia compared to controls (*F* = 5.88, df = 52,1, *p* = 0.019, covaried with RIN). The gene expression of the microglia marker, HEXB, did not show a diagnostic difference that reached the statistical threshold of significance, but was increased at a trend level (*F* = 3.52, df = 50,1, *p* = 0.067, covaried with age and RIN) (Supplementary Figures 1A,B).

**Figure 4:**
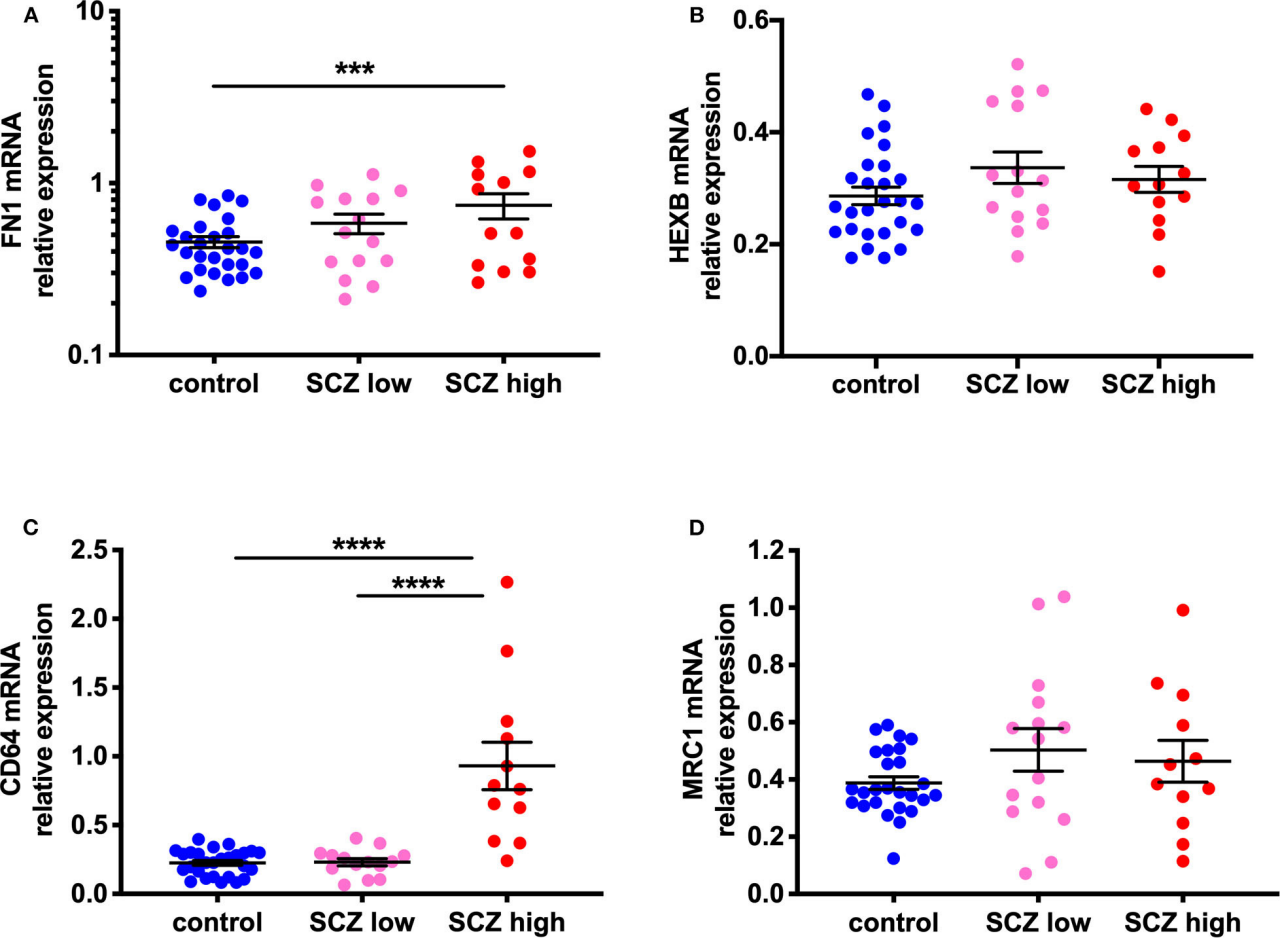
Evidence for increased peripheral macrophages (FN1) and pro-inflammatory potential in the midbrain of a subset of schizophrenia cases classified as high inflammatory. **(A)** Gene expression of the macrophage-enriched marker, fibronectin 1 (FN1; *F* = 4.58, df = 51,2, *p* = 0.015) is increased in the midbrain in schizophrenia cases with a high inflammatory biotype compared to the control group. **(B)** The microglia marker, HEXB mRNA, showed a trend toward a change (*F* = 2.74, df = 50,2, *p* = 0.074). This was due to a small increase (17.57%) in HEXB gene expression in the low inflammatory/schizophrenia subgroup compared to the control group. **(C)** CD64 mRNA, a marker associated with activated or pro-inflammatory macrophages was increased (*F* = 30.19, df = 50,2, *p* < 0.0001) in the high inflammatory/schizophrenia subgroup compared to both the low inflammatory/schizophrenia subgroup and the control group. **(D)** MRC1 mRNA, a marker of anti-inflammatory or resting macrophages, was unchanged by inflammatory subgroup (*F* = 0.11, df = 49,2, *p* = 0.90). Data are mean ± SEM, ***p < 0.001, ****p < 0.0001.

By inflammatory subgroup, FN1 mRNA (*F* = 4.58, df = 51,2, *p* = 0.015) was increased in the midbrain in schizophrenia cases with a high inflammatory biotype (Figure 4A). FN1 mRNA was increased in the high inflammatory/schizophrenia subgroup compared to the control group (63.6%, *p* = 0.004); however, the low inflammatory/schizophrenia subgroup had intermediate FN1 expression. Although the high inflammatory/schizophrenia subgroup had 27.2% more FN1 than the low inflammatory/schizophrenia subgroup, this did not reach statistical significance (*p* = 0.15). Additionally, although the low inflammatory/schizophrenia subgroup had 24.0% more FN1 mRNA than the control group, this also did not reach statistical significance (*p* = 0.161). When comparing the inflammatory subgroups, HEXB mRNA again showed a trend toward an overall change (*F* = 2.74, df = 50,2, *p* = 0.074) (Figure 4B); however, this was due to a small increase (17.6%) in HEXB gene expression in the low inflammatory/schizophrenia subgroup compared to the control group (*p* = 0.025). HEXB gene expression in the high inflammatory/schizophrenia subgroup was not significantly different from the low inflammatory/schizophrenia subgroup or the control group (both *p* > 0.05).

### Evidence for Increased Pro-inflammatory Macrophage Marker, but Not Anti-inflammatory Macrophage Marker

Gene expression of CD64, a marker associated with activated or pro-inflammatory macrophages, was increased (114.98%) in the human midbrain in schizophrenia compared to controls (*t* = −3.33, df = 32.25, *p* = 0.002) (Supplementary Figure 1C). When analyzed by inflammatory subgroup, the high inflammatory/schizophrenia subgroup had ~300% more CD64 mRNA than both the low inflammatory/schizophrenia subgroup and the control group (*F* = 30.19, df = 50,2, *p* < 0.0001, both comparisons *p* < 0.0001) (Figure 4C). The low inflammatory/schizophrenia subgroup was not significantly different compared to the control group (*p* > 0.05).

In contrast, gene expression of MRC1, a marker of anti-inflammatory or resting macrophages, was unchanged by diagnosis (*t* = −0.11, df = 33.20, *p* = 0.91) (Supplementary Figure 1D) or by inflammatory subgroup (*F* = 0.11, df = 49,2, *p* = 0.90) (Figure 4D).

### Gene and Protein Expression of Complement Factors Are Increased in the Midbrain in Schizophrenia Cases With a High Inflammatory Biotype

**Figure 5:**
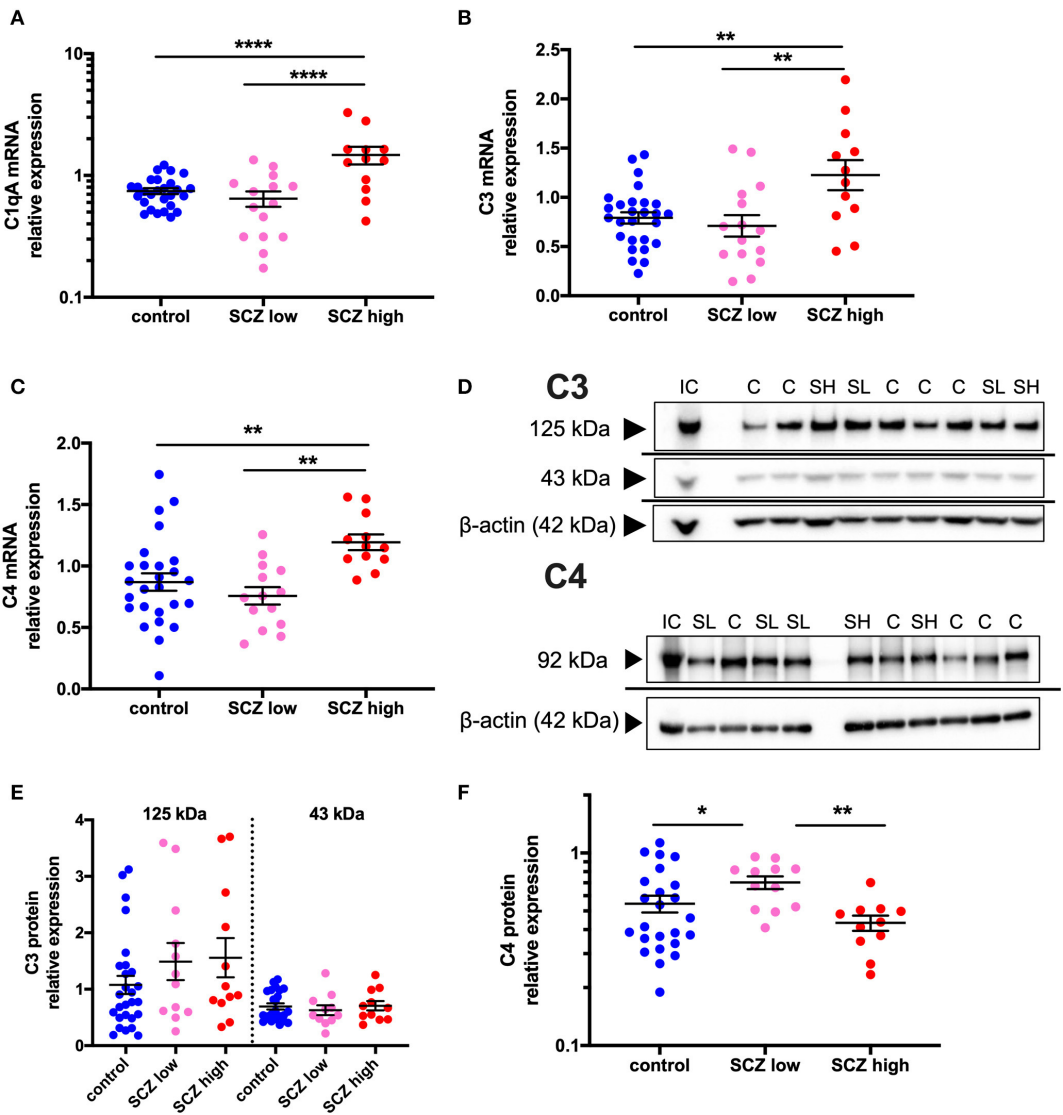
Complement pathway-related transcripts are increased and C4 protein is decreased in the midbrain in schizophrenia cases with a high inflammatory biotype. **(A)** Gene expression of the complement cascade initiator molecule, C1qA, was elevated when analyzed by inflammatory subgroup (*F* = 11.68, df = 50,2, *p* < 0.0001). Gene expression of the complement cascade molecules C3 **(B)** (*F* = 5.51, df = 50,2, *p* = 0.007) and C4 **(C)** (*F* = 6.88, df = 49,2, *p* = 0.002) were also both elevated in the schizophrenia/high inflammatory subgroup compared to the control group and the low inflammatory/schizophrenia subgroup. **(D)** A C4 protein band was detected at 92 kDa and C3 protein bands were detected at 125 and 43 kDa in human midbrain. β-actin (~42 kDa) was detected in all samples and was used as a normalizing control. **(E)** The C3 protein bands were unchanged when analyzed by inflammatory subgroup (all *F* < 1.30, df = 42/43,2, *p* > 0.05). **(F)** C4 protein expression was changed according to inflammatory subgroup (*F* = 4.59, df = 44,2, *p* = 0.015) but C4 protein expression was increased in the low inflammatory/schizophrenia subgroup compared to both the high inflammatory/schizophrenia subgroup and the control group. Data are mean ± SEM, *p < 0.05, **p < 0.01, ****p < 0.0001. C, control; SL, low inflammatory schizophrenia; SH, high inflammatory schizophrenia; IC, internal control.

Gene expression of the complement cascade initiator molecule, C1qA, was not changed by diagnosis (*F* = 0.49, df = 51,1, *p* = 0.49) (Supplementary Figure 2A). However, C1qA mRNA (*F* = 11.68, df = 50,2, *p* < 0.0001) was elevated in the high inflammatory/schizophrenia subgroup compared to the control group (98.2%, *p* ≤ 0.0001) and compared to the low inflammatory/schizophrenia subgroup (128.4%, *p* < 0.0001) (Figure 5A). Neither gene nor protein expression of the complement cascade molecules C3 and C4 were changed by diagnosis: C3 mRNA (*F* = 1.71, df = 51,1, *p* = 0.20, covaried for age); C3 protein levels (125 kDa: *t* = −1.60, 43 kDa: *t* = 0.17; both, df = 48, *p* > 0.05) C4 mRNA (*t* = −0.93, df = 50, *p* = 0.36) and C4 protein levels (*t* = −1.572, df = 49, *p* = 0.122) were unchanged (Supplementary Figures 2B–E).

When analyzing by inflammatory subgroup, C3 mRNA (*F* = 5.51, df = 50,2, *p* = 0.007, covaried for age) (Figure 5B) and C4 mRNA (*F* = 6.88, df = 49,2, *p* = 0.002) (Figure 5C) were both significantly elevated in the high inflammatory/schizophrenia subgroup compared to the control group (54.9% and 37.3%, respectively, both *p* = 0.004), and compared to the low inflammatory/schizophrenia subgroup (72.7%, *p* = 0.004 and 57.6%, *p* = 0.001, respectively). The low inflammatory/schizophrenia subgroup was not significantly different from the control group (*p* > 0.05) for both C3 and C4 mRNAs.

C3 protein bands, corresponding to known molecular weights of the C3 beta subunit and a cleaved C3 beta product at 125 and 43 kDa were detected (51, 52) (Figure 5D). For C4, a 92 kDa band was detected, equivalent to the C4 beta subunit (53, 54) (Figure 5D). Levels of both C3 protein bands were unchanged when analyzed by inflammatory subgroup (125 kDa: *F* = 1.30, df = 46,2, *p* = 0.28; 43 kDa: *F* = 0.29, df = 43,2, *p* = 0.75) (Figure 5E). In contrast, C4 protein expression was altered according to inflammatory subgroup (*F* = 4.59, df = 44,2, *p* = 0.015) but surprisingly, C4 protein was increased in the low inflammatory/schizophrenia subgroup compared to both the high inflammatory/schizophrenia subgroup (62.2%, *p* = 0.005) and compared to the control group (28.8%, *p* = 0.026) (Figure 5F). There was no significant difference in C4 protein levels between the high inflammatory/schizophrenia subgroup and the control group (*p* = 0.23).

### Gene Expression of the “Don't Eat Me” Signals, CD59, but Not CD55, Is Increased in Schizophrenia Cases Exhibiting a High Inflammatory Biotype

**Figure 6:**
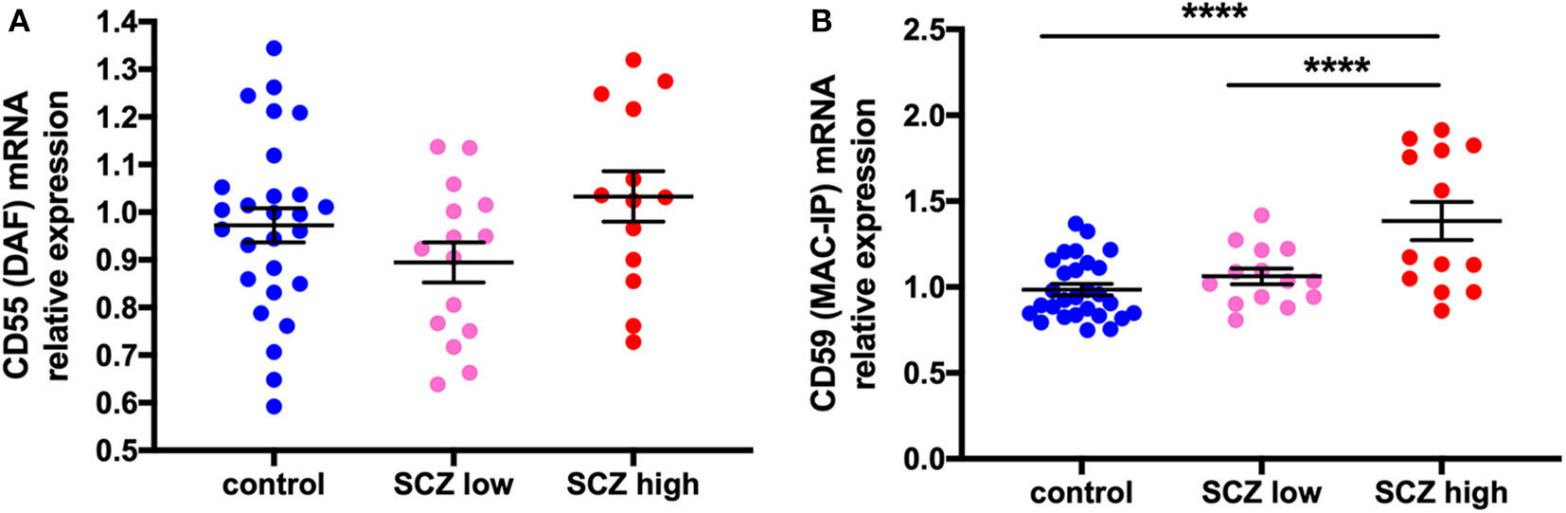
Complement regulator CD59 mRNA, but not CD55 mRNA, is increased in schizophrenia cases with a high inflammatory biotype. **(A)** DAF mRNA was unchanged when analyzed by inflammatory subgroup (*F* = 2.06, df = 52,2, *p* = 0.14). **(B)** In contrast, MAC-IP mRNA was increased when analyzed by inflammatory subgroup (*F* = 16.04, df = 51,2, *p* < 0.0001). Data are mean ± SEM, ****p < 0.0001.

CD55 gene expression was unchanged by diagnosis (*t* = 0.27, df = 53, *p* = 0.79) (Supplementary Figure 2F) or when analyzed by inflammatory subgroup (*F* = 2.06, df = 52,2, *p* = 0.14 (Figure 6A). In contrast, CD59 mRNA was increased by 20.8% in schizophrenia cases compared to controls (*F* = 10.89, df = 53,1, *p* = 0.002) (Supplementary Figure 2G). This was driven by changes due to inflammatory subgroup (*F* = 16.04, df = 51,2, *p* < 0.0001), with an increase in CD59 mRNA in the high inflammatory/schizophrenia subgroup compared to the low inflammatory/schizophrenia subgroup (30.4%) and the control group (40.7%) (both *p* < 0.0001), and no difference between the control group and low inflammatory/schizophrenia subgroup (*p* = 0.23) (Figure 6B).

### C1qA mRNA and Macrophage and Glia Cell Markers Are Positively Associated

**Figure 7:**
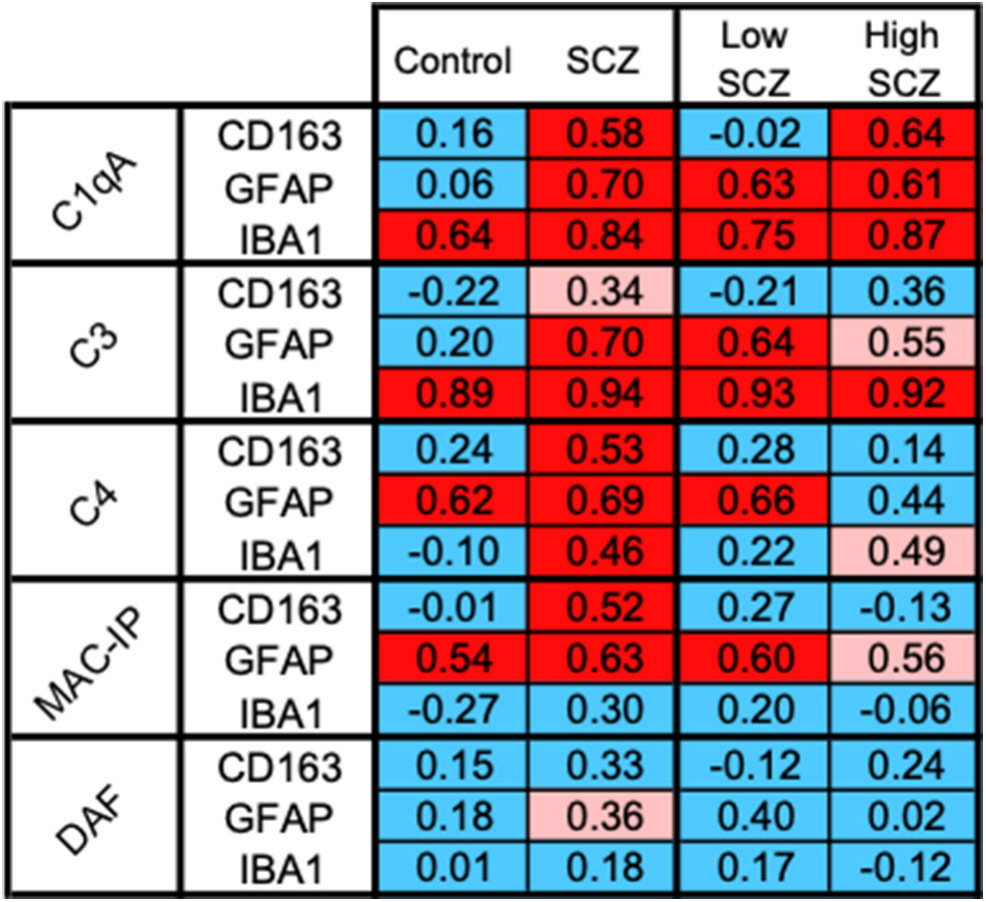
CD163+ cells, the putative macrophages, may be the source of C1qA in the high inflammatory/schizophrenia subgroup. The pattern of positive correlations between CD163 and C1qA is distinguishable from the patterns indicating the relationships between the cell markers and other complement transcripts, suggesting a potentially important relationship between CD163+ infiltrating macrophages and C1qA transcripts in the high inflammatory/schizophrenia group. Blue boxes indicate no significant correlation, pink boxes indicate a trend toward positive correlations, and red boxes indicate positive correlations.

Exploratory correlations between cell markers of putative macrophages (CD163 mRNA), microglia (IBA1 mRNA), and astrocytes (GFAP mRNA) and complement-related transcripts by diagnosis and by inflammatory subgroup indicated that the relationship between C1qA mRNA and CD163 mRNAs was the only one with a distinct effect in the high inflammatory/schizophrenia group. The correlation coefficients between cell markers and complement transcripts are shown in Figure 7, and detailed correlations and statistics are in Supplementary Tables 3A,B.

CD163 mRNA was positively correlated with C1qA mRNA in the schizophrenia cases (red in Figure 7) and not in the control cases (blue in Figure 7), and this correlation was stronger within the high inflammatory/schizophrenia subgroup alone. CD163 mRNA was strongly positively correlated with C1qA mRNA in the high inflammatory/schizophrenia subgroup (*r* = 0.64, *p* = 0.036) (red in Figure 7) but not in the low inflammatory/schizophrenia subgroup or controls (both *r* = −0.02, *p* = 0.95) (blue in Figure 7), and the *z* transformation test comparing the correlations in the schizophrenia inflammatory subgroups trended toward significance (*z* = −1.80, *p* = 0.072).

Of note, the microglia marker transcript, IBA1 mRNA, was strongly positively correlated with C1qA and C3 mRNAs in both the controls and people with schizophrenia overall and when broken down into the two inflammatory/schizophrenia subgroups (all *r* > 0.64, *p* < 0.05) (all red in Figure 7). The astrocyte marker, GFAP mRNA, was strongly positively correlated with C1qA and C3 mRNAs in schizophrenia cases (*r* = 0.71 and 0.70, respectively) but not in control cases (*r* = 0.062 and 0.20, respectively) and these correlations were significantly different when comparing the diagnostic groups (C1qA: *z* = −2.87, *p* = 0.004, and C3: *z* = −2.33, *p* = 0.020, respectively). However, when comparing these correlations within the inflammatory/schizophrenia subgroups, C1qA/GFAP and C3/GFAP were positively correlated in both inflammatory subgroups and these were not significantly different (both *z* < 0.34, *p* > 0.5).

The expression of “don't eat me” signals provides an indication of the protective response against complement-induced damage. CD163 mRNA was strongly positively correlated with CD59 mRNA in schizophrenia cases (*r* = 0.52, *p* = 0.007) but not in controls (*r* = −0.008, *p* = 0.97) and the *z* transformation test was significantly different (*z* = −2.05, *p* = 0.04). CD55 mRNA was not significantly correlated with any complement transcripts in any group, except for a trend toward a positive correlation with GFAP mRNA in schizophrenia (*r* = 0.36, *p* = 0.065).

### Correlations With Antipsychotic Dose Estimates and Midbrain Macrophage and Complement Measurements

Within the schizophrenia group, we tested the relationship between antipsychotic medication and the expression of all mRNA transcripts and levels of proteins measured (Table 2). Positive correlations were detected between CD163, ICAM1, CD64, C1qA, C3, and C4 mRNAs with daily dose of antipsychotics and CD163, ICAM1, and C1qA mRNAs were positively correlated with lifetime levels of antipsychotic exposure (all rho > 0.42, *p* < 0.05). CD163 protein was positively correlated with lifetime exposure (rho = 0.50, *p* = 0.03) and correlated with daily dose of antipsychotics at a trend level of significance (rho = 0.43, *p* = 0.06). Of the complement proteins, only the 125 kDa C3 was negatively correlated with lifetime and mean daily CPZ measures, but this was at a trend level of significance (both rho = 0.44, *p* = 0.07). There were no correlations between midbrain molecular markers and illness duration.

ICAM1 mRNA levels were higher in people who were smokers at the time of death (*t* = 2.85, df = 29.31, *p* = 0.008), whilst CD163, CD59, CD64, and ICAM1 mRNAs were all higher in those that had smoked during their lifetime (*t* < 3.18, df > 34.24, *p* < 0.05). ICAM1 mRNA levels were also increased in patients who reported lifetime depression (*t* = −2.36, df = 49, *p* = 0.022). There were no significant effects of antipsychotic type, suicide, treatment resistance, or positive/negative syndrome on expression of any gene or protein of interest (data not shown).

## Discussion

We provide the first evidence of increased macrophage markers and macrophage density proximal to dopamine cell bodies in schizophrenia, compared to controls, which is especially robust in a subset of schizophrenia cases with a high inflammatory biotype compared to controls (all of which are in the low inflammatory biotype). We provide multiple lines of evidence that are consistent with the possibility that these midbrain macrophages may be infiltrating from the blood into the brain parenchyma. We also provide the first evidence in support of complement activation in the midbrain of people with schizophrenia compared to controls.

The molecular environment in the midbrain in schizophrenia, especially the high inflammatory cases, demonstrates changes that are consistent with increased capture and transmigration of monocytes, as indicated by the increase in ICAM1 mRNA in cases with a high inflammatory biotype. This is similar to the elevation in cortical ICAM1 mRNA, where we localized ICAM1 to the lumen of brain endothelial cells and also identified macrophages in brain tissue in schizophrenia (32). Here, we used CD163 as a marker of peripheral macrophages (33), identified increased CD163 mRNA and protein in midbrain homogenates, and identified an increase in CD163+ cell density and found CD163+ cells that were negative for a common microglial marker. However, microglial-like cells can also be CD163+ (25, 28, 55–57) and determining the lineage of microglia-like cells in the human brain is challenging. In fact, few markers conclusively distinguish between yolk sac-derived (resident microglia) and bone marrow-derived microglia-like cells (perivascular and infiltrating macrophages) (37). Cells expressing IBA1 are generally accepted as microglia; however, IBA1+/CD163+ cells may be microglia upregulating CD163 expression or, alternatively, peripheral macrophages taking on a more microglia-like phenotype once they enter the mammalian brain (37). In our study, we considered CD163+/IBA1- cells to be putative macrophages, and these were identified in the parenchyma. However, we also identified some cells that were CD163+ with light or moderate IBA1 staining intensity, which would be consistent with phenotype switching of either microglial and/or macrophages (37). Thus, these CD163+ cells may represent a variety of cell types or a single cell type at different stages of differentiation. We found that the density of CD163+ cells was significantly increased in the high inflammatory subgroup of people with schizophrenia, as predicted from our homogenate-based measures, suggesting that there may be not only an increase in levels of CD163 per cell but also more CD163+ cells in the midbrain.

Expression of additional microglia- and macrophage- associated transcripts in the midbrain support the idea that macrophages are infiltrating the midbrain parenchyma, along with putative microglial activation *via* increased IBA1 and CD68 mRNAs, previously reported in people with schizophrenia in the high inflammatory subgroup (9). However, HEXB mRNA, which is highly and uniquely expressed in all microglia (including resting), and not expressed by peripheral macrophages, was not significantly changed in those with schizophrenia in our study (35, 36). In contrast, FN1 mRNA, which is highly expressed in peripheral macrophages relative to microglia (35), was increased in the high inflammatory schizophrenia subgroup. This molecular evidence lends support to the suggestion that macrophages may be infiltrating from the blood into the midbrain parenchyma of people with schizophrenia. However, further studies such as single-cell transcriptomics are required to provide more conclusive evidence that these putative macrophages are infiltrating from the periphery.

CD163+ macrophages/microglia are detected in the brain parenchyma in neuroinflammatory diseases and specifically in the substantia nigra in PD, AD, and MS (25, 26, 28, 29). These studies add credence to the possibility that increased CD163+ macrophage infiltration into the midbrain parenchyma may contribute to schizophrenia pathophysiology. Interestingly, once monocytes transmigrate into tissue, they can make and secrete complement (40), and our correlational evidence also suggests that increased macrophage markers appear related to an increase in complement synthesis in the midbrain of people with schizophrenia.

We show, for the first time, increased gene expression of C1qA, C3, and C4 in the midbrain in schizophrenia in cases previously classified as having a high inflammatory status defined by cytokine gene expression (9). This may have been expected as increased pro-inflammatory cytokines and complement expression often co-occur. In fact, increased C4 gene expression in multiple cortical regions in schizophrenia/bipolar cases (48) suggests that there may be wide-spread inflammatory changes in the brain of people with psychosis. Here, we show complement pathway changes are not limited to C4, but involve other key activators (C1qA) and effectors (C3) transcripts of the complement system. However, our findings do not support a straightforward increased level of complement factors in schizophrenia brain, as we do not detect corresponding increases in C3 or C4 complement protein. Contrary to our expectations, C4 protein is significantly decreased in the high inflammatory/schizophrenia subgroup. One interpretation is, if there were increased C3 and C4 proteins as a result of translation of increased complement transcripts, the protein may be rapidly utilized such that the steady state protein levels remain constant (C3) or are reduced (C4) but would still be related to higher levels of transcription.

A more difficult question to consider is “What is the neurobiological consequence of increased complement within the midbrain of a subset of people with schizophrenia?” Complement is linked to synaptic elimination during development and aging (39, 58) and Sekar et al. (48), propose that increased complement in the cortex in schizophrenia may reflect the synaptic loss proposed in the disorder (59). In support of this, they show that C4 protein is associated with neuron cell bodies, processes, and synapses. C1qA and C3 in the brain are also associated with synaptic sculpting (42, 60–62). A recent study found reduced synapse density of inhibitory terminals and a trend toward a reduction in excitatory terminals containing vGlut1 in the midbrain of people with schizophrenia (8). However, it is unclear if the putative increase in complement activity detected here could induce synaptic loss of presynaptic terminals in the adult midbrain. We do see an increase in markers of glial and macrophage cells with phagocytic functions (IBA1, GFAP, and CD163), of phagocytic markers (CD64 and CD68), and increased expression of neuronal “don't eat me” signals (CD59), in the midbrain, in support of increased phagocytosis (9). The particularly strong positive correlation between CD59 and CD163 transcripts in the schizophrenia cases indicates that macrophages are most likely protected from complement-induced damage. Precisely how increased complement, and potentially increased phagocytosis, dysregulate dopamine neurotransmission in the midbrain to potentially contribute to both subcortical hyperdopaminergia and cortical hypodopaminergia remains to be determined.

Another important question that arises from these studies is “Which cells are responsible for the increase in complement transcripts in the midbrain?” Microglia, macrophages, astrocytes, and neurons can all produce complement components (42, 43). Exploratory correlations between control and schizophrenia cases and between the two schizophrenia inflammatory subgroups provided clues to the answer to this question. The relationship between gene expression of C1qA and CD163 reflected a pattern distinct to the high inflammatory/schizophrenia subgroup (Figure 7). In contrast, C1qA mRNA is related to astrocyte and microglia markers in both schizophrenia subgroups irrespective of inflammatory status. Of note, C1qA and C3 mRNAs are strongly related to IBA1 mRNA irrespective of diagnosis or inflammatory state (as indicated by four red boxes in Figure 7), suggesting that microglia and C1qA and C3 are closely linked in general and these complement components, insofar as they are related to “normal” microglia, are unlikely to play an exaggerated role in the disease process where more “activated” microglia appear. With respect to astrocytes, the correlation between a reactive astrocyte marker and complement in the midbrain in schizophrenia suggested increased complement in astrocytes with more severe inflammatory changes in the disease, but this is not unique to the high inflammatory subgroups as is the relationship between C1qA and the macrophage marker. We acknowledge that these are correlative studies and the schizophrenia inflammatory subgroups reduce statistical power (~14 cases). Nonetheless, they suggest that CD163+ cells are possibly salient players in the initiation of the complement cascade in cases with a high inflammatory biotype. Single cell transcriptomic studies of macrophage populations isolated from the midbrain are required to test this hypothesis. Although our evidence points toward the putative infiltrating macrophages as important, it also suggests that they work in concert with other resident glial cells to execute the full complement activation within the human midbrain.

A key protective mechanism utilized by neurons to withstand complement activation and to survive an inflammatory insult is the “don't eat me” signal or CD55 expression (63). Chronic experimental autoimmune encephalitis in marmoset is associated with a robust increase in neuronal expression of CD55 (63). The lack of increased CD55 transcripts in the midbrain in schizophrenia cases with a high inflammatory biotype suggests the neurons may not be mounting an effective defense against complement-induced damage (potentially synaptic elimination), or that this protective mechanism does not work in the expected way in the human midbrain. Conversely, CD59, which inhibits the formation of the membrane attack complex and cell lysis (49) and can also be considered a “don't eat me” signal, is increased in the high inflammatory schizophrenia cases. This implies that cells, potentially including neurons, could be mounting an attempt at protection from complement-induced cell lysis/death as CD59 in neurons in the brain is related to the need for protection against complement in AD and following traumatic brain injury (64–66).

Interestingly, the immune activation in the midbrain appears to be of a greater magnitude and more encompassing than we detect in the cortex. In the midbrain, we find a greater proportion of cases being classified as high inflammatory, we find greater levels of inflammatory changes overall, and we report evidence of robust astrogliosis and increased microglia (9) and, herein, evidence of a greater increase in macrophages even when comparing by diagnostic groups alone. In contrast, in the cortex, we detect evidence of astrogliosis and increased infiltrating macrophages only when analyzing by inflammatory subgroup and not when analyzing by diagnostic groups (12, 13, 32). The reason why there may be greater inflammatory-related neuropathology within the midbrain compared to the cortex is not clear. We have explored some clinical factors and found that smoking did appear to influence gene expression of multiple mRNA markers (ICAM1, CD163, CD59, and CD64) in the midbrain and this needs to be considered as a caveat when interpreting the results. Interestingly, no other clinical (treatment resistance, class of antipsychotic) or ante-mortem (suicide) factors were associated with any of the markers measured. However, our study identified positive correlations of CD163 mRNA/protein levels with antipsychotic levels. Our study also identified strong positive correlations between antipsychotic exposure and multiple complement transcripts (C1qA, C3, and C4) but no relationship with complement regulator transcripts (CD55 and CD59). Although this may indicate that antipsychotics increased putative macrophages and induce complement, there is also data to suggest that antipsychotics dampen inflammation (21). Additionally, complement system activity is increased in first onset schizophrenia patients (67, 68), and in antipsychotic-naïve or antipsychotic-free schizophrenia patients (68). There is some evidence from pre-clinical rodent models to suggest that increased complement in the brain may be more directly related to the disease process. A study in maternal immune activation (MIA) exposed rats found increased cortical C4 transcripts without antipsychotic treatment (69). We have previously reported that the high inflammatory schizophrenia group received substantially higher doses of antipsychotics than the cases in the schizophrenia/low inflammatory subgroup during life (9). We acknowledged that this may contribute to the greater inflammatory-related gene expression changes, however, we also suggested that it may reflect that the individual with high neuroinflammation within the midbrain has more severe symptoms that require a higher daily dose of antipsychotic medications to be managed (9). More studies on the consequences of antipsychotics on midbrain neuroinflammation are needed to address if increases in gliosis, cytokines, and complement could be caused by long-term antipsychotic treatment.

There are other aspects of increased neuroinflammation and complement in the midbrain in schizophrenia that should also be considered. Activation of the complement pathway in the midbrain may be due to the presence of autoantibodies. Although we found similar levels of IgG antibodies in the prefrontal cortex of control and schizophrenia cases (70), investigating the presence of autoantibodies in the midbrain in schizophrenia is warranted because antibodies targeting midbrain dopamine neurons would be expected to be concentrated in the midbrain and neuroinflammation in the midbrain appears to be greater than what we see in the cortex (12). A further aspect that we have not explored in this work is the dopaminergic regulation of the immune system. Immune cells, including *T* and *B* lymphocytes, dendritic cells, natural killer cells, macrophages, and microglia, express dopamine receptors and thus increased dopamine, as proposed to occur in schizophrenia, may itself regulate inflammatory activity [reviewed in (71)]. Indeed, studies have identified *B* and *T* cells in the cortex and hippocampus of some people with schizophrenia (72, 73), and investigating the presence of other peripheral immune cells in the midbrain and how these cells may be influenced by changes to dopamine or with antipsychotics in schizophrenia, is of interest. Evidence of peripheral immune cells infiltrating the brain parenchyma suggests disruption of the blood-brain barrier (BBB) in schizophrenia [reviewed in (74, 75)]. As in the cortex in schizophrenia (32), here we report increased ICAM1 mRNA in the midbrain associated with neuroinflammation in schizophrenia, indicating that molecular changes in the BBB exist. Although we have not found direct evidence for transcriptional changes consistent with a leaky BBB in schizophrenia (32), others report that claudin-5 protein, a tight junction protein, may be reduced in schizophrenia (76, 77). Interestingly, reduced claudin-5 in mice induces psychosis-like behaviors (77). Further investigation of the status of the BBB, including tight junctions, in the midbrain in schizophrenia may provide support for the infiltration of peripheral immune cells, including monocytes. Immune cells are attracted to sites of damage or pathology by mediators such as cytokines and chemokines, for example, IL8, monocyte chemoattractant protein-1 (MCP-1 or CCL2), macrophage inflammatory protein-1 alpha (MIP-1 alpha/CCL3) or MIP-1 beta (CCL4) (78–82). Interestingly, IL8 was not increased in the midbrain in schizophrenia (9), suggesting that other macrophage chemoattractant transcripts may be increased or that IL8 may be changed at the protein level. Further studies to determine which molecules may be the potential mediators of the putative increased attraction and entry of macrophages to the midbrain parenchyma in schizophrenia are necessary.

In conclusion, we provide the first evidence to suggest that macrophage or macrophage-like cells may be a component of the neuropathology within the midbrain in schizophrenia, especially those cases with a high inflammatory signature. We also provide the first evidence that complement transcripts are increased in the midbrain in schizophrenia compared to controls, again especially in those with high cytokine transcript levels. Our work adds to the growing body of evidence that neuroinflammation is evident in a subset of people with schizophrenia and supports that multiple inflammatory pathways are increased in multiple brain regions, including the midbrain. We show that the human midbrain may be especially sensitive to inflammatory pathology in schizophrenia, but linking these findings to the known dopamine dysregulation, determining the inflammatory effects of antipsychotics, and using these findings to inform the identification of novel treatments are current challenges in our field.

## Data Availability Statement

The raw data supporting the conclusions of this article will be made available by the authors, without undue reservation, to any qualified researcher.

## Ethics Statement

The studies involving human participants were reviewed and approved by University of New South Wales Human Research Ethics Committee. Written informed consent for participation was not required for this study in accordance with the national legislation and the institutional requirements.

## Author Contributions

TPT and CSW contributed to study design and conception, interpretation of results and edited the manuscript. DB and AB performed qPCRs. KR performed CD163 immunohistochemistry and cell counts. HC, DB, and AB contributed to CD163, C3 and C4 immunoblotting. CW performed the IBA1/CD163 fluorescence immunohistochemistry. KR, AB, DB, SO, and DR contributed to statistical analysis. TPT contributed to statistical analysis and wrote the manuscript. All authors edited the manuscript.

## Conflict of Interest

CSW is on an advisory board for Lundbeck, Australia Pty Ltd and in collaboration with Astellas Pharma Inc., Japan. The remaining authors declare that the research was conducted in the absence of any commercial or financial relationships that could be construed as a potential conflict of interest.
