# Supplementary material for: Increased Macrophages and C1qA, C3, C4 Transcripts in the Midbrain of People With Schizophrenia
Source: Front Immunol. 2020 Sep 29;11:2002. doi: 10.3389/fimmu.2020.02002 (PMC7550636; doi:10.3389/fimmu.2020.02002)
Supplement: Supplementary file 1 [file Table_1.docx]

**Supplementary Table 1**: Taqman gene expression assays (Life Technologies, Australia)

| Transcript | Taqman probe | Transcript | Taqman probe |
| --- | --- | --- | --- |
| β-actin | Hs99999903_m1 | **CD59** | Hs00174141_m1 |
| TBP | Hs00427620_m1 | **ICAM1** | Hs00164932_m1 |
| GAPDH | Hs99999905_m1 | **CD163** | Hs00174705_m1 |
| UBC | Hs00824723_m1 | **MRC1** | Hs00267207_m1 |
| C1QA | Hs00381122_m1 | **HEXB** | Hs01077594_m1 |
| C3 | Hs00163811_m1 | **CD64** | Hs00174081_m1 |
| C4 | Hs00246758_m1 | **FN1** | Hs01549976_m1 |
| CD55 | Hs00892618_m1 |  |  |
