# Supplementary material for: Increased Macrophages and C1qA, C3, C4 Transcripts in the Midbrain of People With Schizophrenia
Source: Front Immunol. 2020 Sep 29;11:2002. doi: 10.3389/fimmu.2020.02002 (PMC7550636; doi:10.3389/fimmu.2020.02002)
Supplement: Supplementary file 2 [file Table_2.docx]

**Supplementary Table 2:** Correlations between macrophage marker and complement

gene and protein expression and age at death, brain pH, RIN, and PMI.

| Gene/protein of  interest | Covariate | N | Correlation  coefficient | *p* |
| --- | --- | --- | --- | --- |
| CD163 mRNA | Age  pH  RIN  PMI | 54  54  54  54 | **0.292**  **-0.413**  -0.258  0.240 | **0.036***  **0.002****  0.065  0.086 |
| ICAM mRNA | Age  pH  RIN  PMI | 54  54  54  54 | 0.182  **-0.394**  -0.263  0.107 | 0.187  **0.003*****  0.055#  0.440 |
| CD64 mRNA | Age  pH  RIN  PMI | 53  53  53  53 | 0.155  -0.050  0.110  0.148 | 0.268  0.724  0.433  0.289 |
| MRC1 mRNA | Age  pH  RIN  PMI | 52  52  52  52 | 0.168  -0.201  -0.164  -0.162 | 0.229  0.149  0.242  0.246 |
| FN1 mRNA | Age  pH  RIN  PMI | 54  54  54  54 | 0.058  **-0.455**  **-0.494**  -0.224 | 0.679  **0.001*****  **<0.0001******  0.104 |
| HEXB mRNA | Age  pH  RIN  PMI | 54  54  54  54 | **0.325**  **-0.391**  **-0.571**  0.197 | **0.017***  **0.003*****  **<0.0001******  0.154 |
| C1qA mRNA | Age  pH  RIN  PMI | 54  54  54  54 | 0.249  -0.014  -0.077  0.030 | 0.069  0.922  0.579  0.827 |
| C3 mRNA | Age  pH  RIN  PMI | 54  54  54  54 | **0.302**  0.165  0.115  0.131 | **0.027***  0.234  0.409  0.344 |
| C4 mRNA | Age  pH  RIN  PMI | 52  52  52  52 | -0.049  -0.267  -0.051  0.100 | 0.731  0.056  0.721  0.479 |
| CD59 mRNA | Age  pH  RIN  PMI | 56  56  56  56 | 0.221  **-0.497**  **-0.441**  0.214 | 0.101  **<0.0001******  **0.001****  0.113 |
| CD55 mRNA | Age  pH  RIN  PMI | 55  55  55  55 | 0.214  -0.091  0.042  -0.071 | 0.116  0.507  0.761  0.608 |
| CD163 protein | Age  pH  PMI | 52  52  52 | 0.269  **-0.328**  0.253 | 0.054#  **0.018***  0.070# |
| C3 protein  125 kDa | Age  pH  PMI | 51  51  51 | -0.087  -0.150  0.000 | 0.542  0.293  0.999 |
| C3 protein  43 kDa | Age  pH  PMI | 49  49  49 | 0.137  -0.263  0.202 | 0.348  0.068^#^  0.165 |
| C4 protein  92 kDa | Age  pH  PMI | 49  49  49 | -0.142  -0.083  -0.078 | 0.332  0.571  0.594 |

Bold values denote significant correlations. ^#^*p* < 0.1 **p* < 0.05, ***p* < 0.01, ****p* < 0.001, *****p* < 0.0001.
