# Supplementary material for: Increased Macrophages and C1qA, C3, C4 Transcripts in the Midbrain of People With Schizophrenia
Source: Front Immunol. 2020 Sep 29;11:2002. doi: 10.3389/fimmu.2020.02002 (PMC7550636; doi:10.3389/fimmu.2020.02002)
Supplement: Supplementary file 3 [file Table_3.docx]

**Supplementary Table 3A**. Correlations between complement-related and cell marker gene expression in control and schizophrenia cases

|  | controls | | | schizophrenia | | | Fischer’s r to Z | | |
| --- | --- | --- | --- | --- | --- | --- | --- | --- | --- |
|  | r | *p*-value | N | r | *p*-value | N | | *Z* | *p*-value |
| CD163 vs C1qA | 0.157 | 0.443 | 28 | 0.576 | **0.002***** | 28 | | -1.76 | 0.078# |
| CD163 vs C3 | -0.215 | 0.293 | 28 | 0.342 | 0.087**#** | 28 | | -2.03 | **0.042*** |
| CD163 vs C4 | 0.235 | 0.248 | 28 | 0.531 | **0.005**** | 28 | | -1.24 | 0.215 |
| CD163 vs CD59 | -0.008 | 0.968 | 28 | 0.517 | **0.007**** | 28 | | -2.05 | **0.040*** |
| CD163 vs CD55 | 0.152 | 0.458 | 28 | 0.325 | 0.106 | 28 | | -0.65 | 0.516 |
| IBA1 vs C1qA | 0.641 | **<0.0001**  ******** | 28 | 0.844 | **<0.0001**  ******** | 28 | | -1.68 | 0.093**#** |
| IBA1 vs C3 | 0.886 | **<0.0001**  ******** | 28 | 0.936 | **<0.0001**  ******** | 28 | | -1.07 | -0.285 |
| IBA1 vs C4 | -0.099 | 0.615 | 28 | 0.456 | **0.015*** | 28 | | -2.09 | **0.037*** |
| IBA1 vs CD59 | -0.271 | 0.172 | 28 | 0.302 | 0.126 | 28 | | -2.08 | **0.038*** |
| IBA1 vs CD55 | 0.009 | 0.964 | 28 | 0.184 | 0.348 | 28 | | -0.63 | 0.529 |
| GFAP vs C1qA | 0.062 | 0.758 | 28 | 0.703 | **<0.0001**  ******** | 28 | | -2.87 | **0.004**  ******* |
| GFAP vs C3 | 0.196 | 0.327 | 28 | 0.695 | **<0.0001**  ******** | 28 | | -2.33 | **0.020*** |
| GFAP vs C4 | 0.616 | **0.001**  ******* | 28 | 0.688 | **<0.0001**  ******** | 28 | | -0.44 | 0.660 |
| GFAP vs CD59 | 0.54 | **0.004*** | 28 | 0.628 | **<0.0001**  ******** | 28 | | -0.47 | 0.638 |
| GFAP vs CD55 | 0.177 | 0.376 | 28 | 0.36 | 0.065# | 28 | | -0.70 | 0.484 |

Bold values denote significant correlations. ^#^*p* < 0.1 **p* < 0.05, ***p* < 0.01, ****p* < 0.001, *****p* < 0.0001.

**Supplementary Table 3B.** Correlations between complement-related and cell marker gene expression in high and low immune schizophrenia case

|  |  | | Schizophrenia/low | | | Schizophrenia/high | | | Fischer’s r to Z | |
| --- | --- | --- | --- | --- | --- | --- | --- | --- | --- | --- |
|  | | r | | *p*-value | N | r | *p*-value | N | Z | *p*-value |
| CD163 vs C1qA | | -0.018 | | 0.953 | 15 | 0.636 | **0.036*** | 13 | -1.80 | 0.072**#** |
| CD163 vs C3 | | -0.210 | | 0.491 | 15 | 0.357 | 0.281 | 13 | -1.37 | 0.171 |
| CD163 vs C4 | | 0.281 | | 0.353 | 15 | 0.140 | 0.682 | 13 | 0.35 | 0.726 |
| CD163 vs CD59 | | 0.266 | | 0.380 | 15 | -0.126 | 0.712 | 13 | 0.93 | 0.352 |
| CD163 vs CD55 | | -0.117 | | 0.704 | 15 | 0.244 | 0.469 | 13 | -0.86 | 0.390 |
| IBA1 vs C1qA | | 0.745 | | **<0.001***** | 15 | 0.865 | **<0.0001****** | 13 | -0.82 | 0.412 |
| IBA1 vs C3 | | 0.931 | | **<0.0001****** | 15 | 0.922 | **<0.0001****** | 13 | 0.15 | 0.881 |
| IBA1 vs C4 | | 0.222 | | 0.426 | 15 | 0.492 | 0.087**#** | 13 | -0.73 | 0.465 |
| IBA1 vs CD59 | | 0.201 | | 0.490 | 15 | -0.059 | 0.855 | 13 | 0.61 | 0.542 |
| IBA1 vs CD55 | | 0.17 | | 0.544 | 15 | -0.115 | 0.709 | 13 | -0.67 | 0.503 |
| GFAP vs C1qA | | 0.633 | | **0.015*** | 15 | 0.608 | **0.036*** | 13 | 0.09 | 0.928 |
| GFAP vs C3 | | 0.642 | | **0.013*** | 15 | 0.548 | 0.065**#** | 13 | 0.34 | 0.734 |
| GFAP vs C4 | | 0.658 | | **0.011*** | 15 | 0.44 | 0.153 | 13 | 0.74 | 0.459 |
| GFAP vs CD59 | | 0.599 | | **0.024*** | 15 | 0.559 | 0.059**#** | 13 | 0.14 | 0.889 |
| GFAP vs CD55 | | 0.403 | | 0.153 | 15 | 0.015 | 0.964 | 13 | 0.96 | 0.337 |

Bold values denote significant correlations. ^#^*p* < 0.1 **p* < 0.05, ****p* < 0.001, *****p* < 0.0001.
