# Supplementary material for: Increased Macrophages and C1qA, C3, C4 Transcripts in the Midbrain of People With Schizophrenia
Source: Front Immunol. 2020 Sep 29;11:2002. doi: 10.3389/fimmu.2020.02002 (PMC7550636; doi:10.3389/fimmu.2020.02002)
Supplement: Supplementary file 4 [file Image_1.pdf]

Supplementary Figure 1

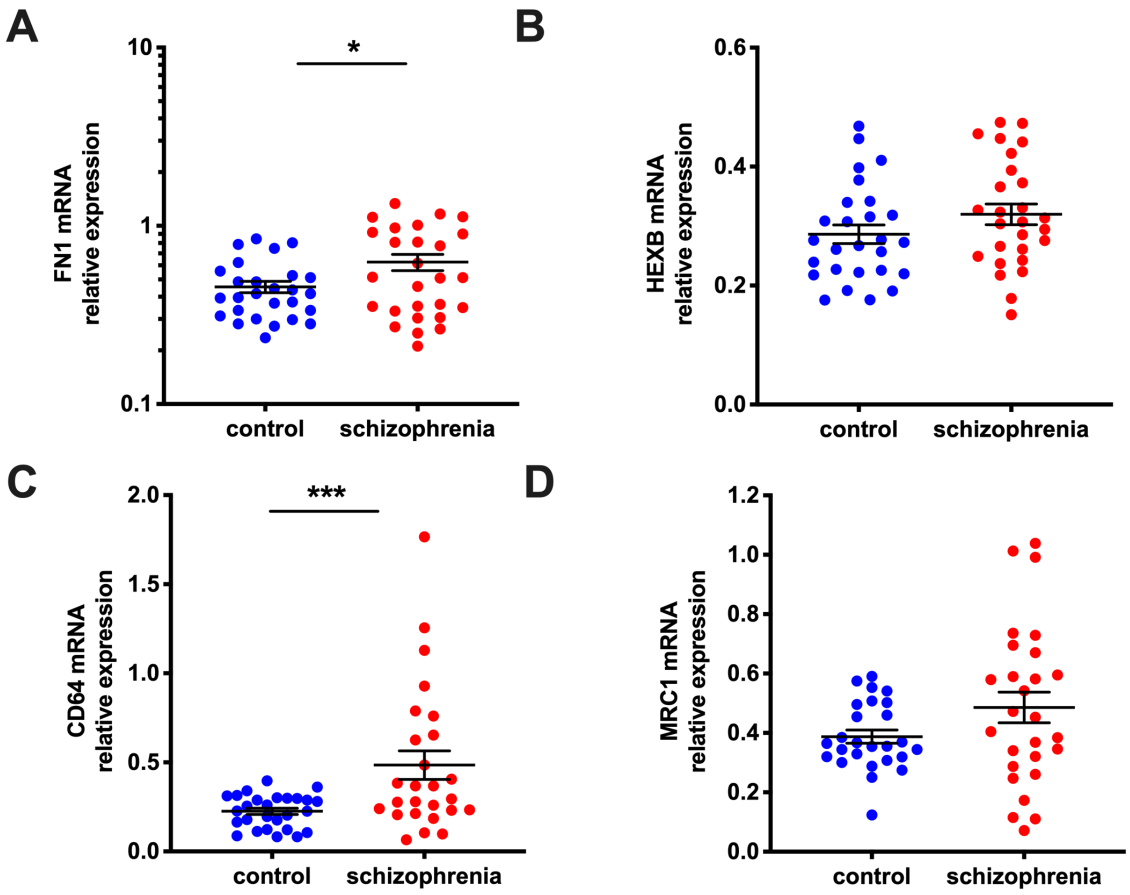

Supplementary Figure 1. Evidence for increased peripheral macrophages (FN1) and pro-inflammatory potential (CD64 mRNA) in the midbrain of schizophrenia compared to controls. (A) FN1 mRNA, expressed by peripheral macrophages and not microglia, was increased in the midbrain in schizophrenia compared to controls (F= 5.88, df = 52,1, p = 0.019, covaried with RIN). (B) HEXB mRNA, a microglia marker, trended towards an increase when analysed by diagnosis (F= 3.52, df = 50,1, p = 0.067, covaried with age and RIN). (C) CD64 mRNA, a marker associated with activated or pro-inflammatory macrophages was increased (114.98%) in the human midbrain in schizophrenia compared to controls (t = -3.33, df = 32.25, p = 0.002). (D) MRC1 mRNA, a marker of anti-inflammatory or resting macrophages, was unchanged by diagnosis (t = -0.78, df = 35.11, p = 0.44). Data are mean ± SEM, * p< 0.05, *** p< 0.001.
