# Supplementary material for: Increased Macrophages and C1qA, C3, C4 Transcripts in the Midbrain of People With Schizophrenia
Source: Front Immunol. 2020 Sep 29;11:2002. doi: 10.3389/fimmu.2020.02002 (PMC7550636; doi:10.3389/fimmu.2020.02002)
Supplement: Supplementary file 5 [file Image_2.pdf]

Supplementary Figure 2

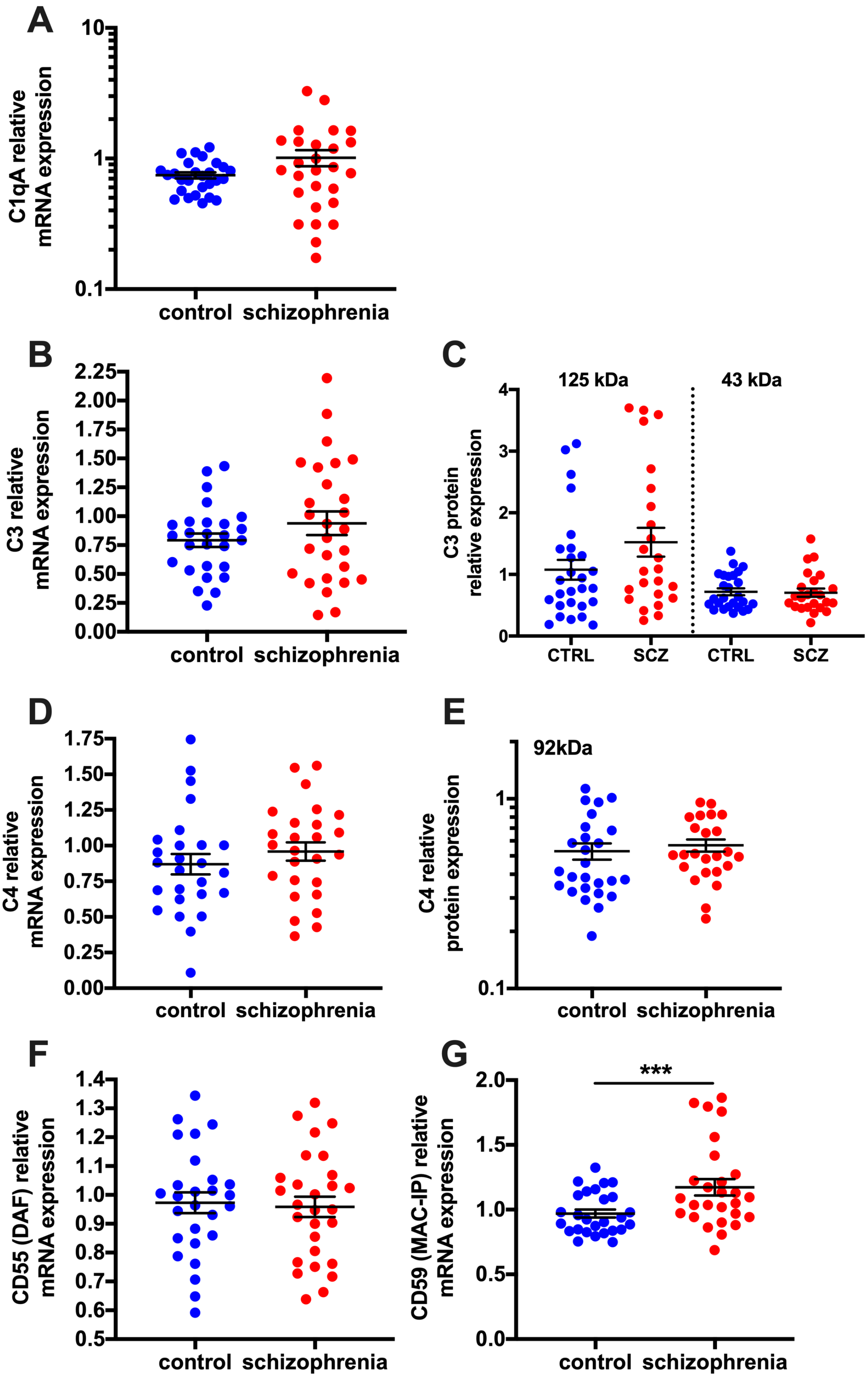

Supplementary Figure 2. Complement pathway-related transcripts and protein in the midbrain in schizophrenia cases and controls. (A) Gene expression of the complement cascade initiator molecule, C1qA was not changed by diagnosis (F = 0.49, df=51,1, $p = 0.49$). (B) C3 mRNA (F=1.71, df=51,1, $p = 0.20$, covaried for age) and (C) C3 protein levels (125 kDa: $t = -1.57$, df = 49; 43 kDa: $t = -0.033$, df = 47, both $p > 0.05$) were unchanged by diagnosis. (D) C4 mRNA ($t = -0.93$, df = 50, $p = 0.36$) and (E) C4 protein levels ($t = -1.57$, df = 49, $p = 0.12$) were unchanged by diagnosis. (F) CD55 mRNA was unchanged by diagnosis ($t = 0.27$, df = 53, $p = 0.79$) and (G) CD59 was increased in schizophrenia cases compared to controls (F = 10.89, df = 53,1, $p = 0.002$). Data are mean ± SEM, *** $p < 0.001$.
